# A Donor Registry: Genomic Analyses of *Posidonia australis* Seagrass Meadows Identifies Adaptive Genotypes for Future‐Proofing

**DOI:** 10.1002/ece3.70667

**Published:** 2024-12-06

**Authors:** Matt J. Nimbs, Tim M. Glasby, Elizabeth A. Sinclair, Daniel Swadling, Tom R. Davis, Melinda A. Coleman

**Affiliations:** ^1^ Fisheries Research, New South Wales Department of Primary Industries and Regional Development National Marine Science Centre Coffs Harbour New South Wales Australia; ^2^ National Marine Science Centre Southern Cross University Coffs Harbour New South Wales Australia; ^3^ Fisheries Research, New South Wales Department of Primary Industries and Regional Development Port Stephens Fisheries Research Institute Taylors Beach New South Wales Australia; ^4^ School of Biological Science and Oceans Institute The University of Western Australia Nedlands Western Australia Australia

**Keywords:** climate change, estuaries, genotype environment associations, gradient forest, single‐nucleotide polymorphisms

## Abstract

Globally, anthropogenic climate change has caused declines of seagrass ecosystems necessitating proactive restoration approaches that would ideally anticipate future climate scenarios, such as marine warming. In eastern Australia, estuaries with meadows of the endangered seagrass *Posidonia australi*s have warmed and acidified over the past decade, and seagrass communities have declined in some estuaries. Securing these valuable habitats will require proactive conservation and restoration efforts that could be augmented with restoration focussed on boosting resilience to future climate. Understanding patterns of selection and where seagrass meadows are adapted to particular environmental conditions is key for identifying optimal donor material for restoration. We used single nucleotide polymorphisms and genotype by environment analyses to identify candidate loci under putative selection to environmental stressors and assess genomic variation and allelic turnover along stressor gradients. The most important physicochemical variables driving selection were associated with temperature, water turbidity, and pH. We developed a preliminary ‘donor registry’ of pre‐adapted *P. australis* genotypes by mapping the distribution of alleles to visualise allelic composition of each sampled seagrass meadow. The registry could be used as a first step to select source material for future‐proofing restoration projects. A next step is to establish manipulative experiments that will be required to test whether pre‐adapted genotypes confer increased resistance to multiple environmental stressors.

## Graphical abstract

Genotype–environmental analyses were used to identify candidate single nucleotide polymorphisms, which were used to map allelic turnover along estuarine environmental gradients. Visualisation of allelic distributions were then used to identify the location of genotypes that are potentially pre‐adapted to certain environmental conditions, which can be analogous to future environment in other locations—a donor registry.

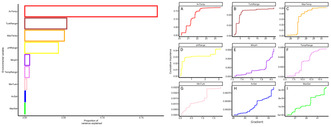

## 1 Introduction

The highly dynamic nature of estuarine systems means that climate change‐driven trends are more difficult to identify than in ocean systems (Dyer 2021). There continues to be mounting evidence of warming, particularly in large riverine and bay estuaries on a global scale (Ashizawa and Cole 1994; Marbà and Duarte 2010; Oczkowski et al. 2015; Wiltshire and Manly 2004). In particular, the coast of south‐eastern Australia is a climate change hotspot with warming occurring more rapidly (> 90% faster) than global averages (Hobday and Pecl 2014; Fulton et al. 2024). Estuaries in New South Wales (NSW) have exhibited rapid environmental change, warming at 0.2°C per year over the last 12 years, whilst acidity has increased (lower pH) and salinity has tended to decrease in some types of small estuaries (Scanes, Scanes, and Ross 2020).

Estuaries support numerous biotic habitats, including seagrass meadows, which are often found in shallow, low‐energy systems (den Hartog and Kuo 2006; Hogarth 2007; Inglis and Smith 1998; Unsworth, Nordlund, and Cullen‐Unsworth 2019). Globally, there have been reductions in seagrass extent and increases in fragmentation due to a range of human activities related to the urbanisation of coastal areas, including eutrophication, altered sediment dynamics, and mechanical destruction (Grech et al. 2012; Waycott et al. 2009; Dunic et al. 2021; Swadling et al. 2023a). Seagrass meadows are also increasingly suffering the deleterious effects of climate change, including warming, marine heatwaves, sea level rise, and altered storm patterns (Duarte et al. 2018; Guerrero‐Meseguer et al. 2020; Strydom et al. 2020). Climate change stressors such as increased temperatures may also affect the reproductive timing and output of seagrasses (Díaz‐Almela et al. 2009; Ruocco et al. 2022; Lekammudiyanse et al. 2024; Tomas et al. 2024) and, crucially, may exacerbate many of the pre‐existing direct anthropogenic impacts (McMahon et al. 2022). Equatorial range‐edge populations remain particularly vulnerable to climate change as the planet warms (Bartenfelder et al. 2022). In these locations, it has been suggested that conservation activities such as meadow restoration and assisted adaptation should be a high priority (Pazzaglia et al. 2021a).

An important biological consideration for successful seagrass meadow restoration is establishing and maintaining genetic diversity to boost resilience (Evans et al. 2018; Procaccini and Piazzi 2001; Reynolds, McGlathery, and Waycott 2012). However, there is increasing acknowledgement that contemporary restoration methods should also consider climate‐adjusted provenancing (Prober et al. 2015; Wood et al. 2024) to help future‐proof restored seagrass meadows (Wood et al. 2019; Tan et al. 2020; Pazzaglia et al. 2021b). Climate‐adjusted provenancing involves identifying donor meadows that are genetically pre‐adapted to particular environmental conditions, such as warmer temperatures, meaning that they will be more likely to survive under projected environmental change (Nguyen et al. 2023; Tan et al. 2020; Krauss et al. 2013). Such projects can include assisted adaptation and/or assisted migration and generally rely on introducing genotypes that are adapted to future conditions, such as elevated temperatures. Suitable genotypes can be found in areas that have pre‐adapted to environmental conditions that are already similar to those predicted to characterise oceans and estuaries in the future (Krauss et al. 2013; Prober et al. 2019; Webster et al. 2023). A prerequisite for applying climate provenance techniques as part of restoration program planning is the identification of pre‐adapted standing genetic diversity among populations within the species native range. Climate‐adjusted provenancing is increasingly recommended as an optimal approach in marine (Coleman et al. 2020), and terrestrial systems (Benito‐Garzón et al. 2013; Winder, Nelson, and Beardmore 2011), however there remains a paucity of studies regarding the development, roll‐out, and success or otherwise of this approach in seagrass and other systems.

**FIGURE 1:**
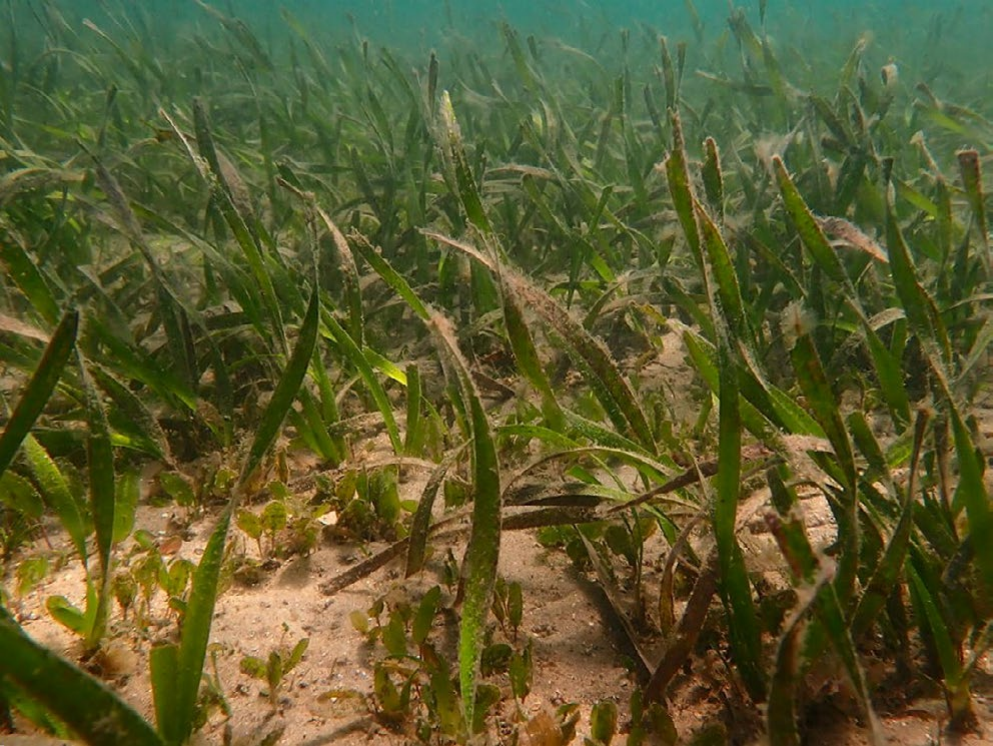
*Posidonia australis* meadow, Fly Point, Port Stephens, New South Wales. Photo: MJN.

The broad‐leaved seagrass, *P. australis* J. D. Hooker, 1858, is the only species in the genus *Posidonia* found in NSW (Figure 1). It occurs in 17 coastal estuaries and a small number of sheltered coastal locations between 32° S to 37° S (Scanes, Scanes, and Ross 2020; West and Glasby 2022; West, Larkum, and King 1989). The species has been listed as endangered in six NSW estuaries due to a range of historical and contemporary anthropogenic impacts which can vary greatly among and within estuaries (West and Glasby 2022; Swadling et al. 2023b) and which have resulted in reductions in extent and increased fragmentation of meadows (Swadling et al. 2023a). All *P. australis* meadows on the Manning‐Hawkesbury shelf are federally listed as endangered in 2015 under the EPBC Act (Commonwealth of Australia). *P. australis*, being a long‐lived (persistent) species is slow to recover from disturbances due to its slow growth rate, variable sexual reproduction and lack of seed dormancy (Kilminster et al. 2015). In NSW, the distribution of *P. australis* across a latitudinal gradient and different geomorphic types of estuaries (West and Glasby 2022) means that populations in different estuaries may be subjected to different environmental conditions, either historically, or currently and into the future due to climate change. Phenotypic response to environmental conditions in *P. australis* is governed by its inherent genomic characteristics and population dynamics (Duarte et al. 2018). Given that NSW estuaries have become warmer, fresher (lower salinity), and more acidic (lower pH) over the past two decades (Scanes, Scanes, and Ross 2020), the need to study genomic variation and functional response, identify standing adaptive potential, and explore potential resilience is more important than ever. This may be particularly salient among the northern, range‐edge populations, which, due to their genetic isolation (Evans et al. 2014) and exposure to higher temperatures, may be under imminent threat (Duarte et al. 2018).

The genetic structure of *P. australis* populations along the NSW coast shows a highly significant positive relationship between clonal diversity, allelic diversity, and heterozygosity with increasing latitude (Evans et al. 2014; Sinclair et al. 2023). Some populations reflect an ancestral history of geographic isolation associated with the effects of sea level rise after the last glacial maxima, the presence of shared multilocus genotypes amongst present‐day geographically isolated populations in the northern range is consistent with historical connectivity (Evans et al. 2014; Sinclair et al. 2023). Given the very low chances of natural gene flow amongst modern‐day estuaries, this indicates the presence of a common ancestral source meadow. Significant population differentiation among meadows in southern NSW appears to be associated with the 14°C sea surface isotherm (Evans et al. 2014; Sinclair et al. 2023). Here, we identify seagrass meadows that support *P. australis* genotypes pre‐adapted to various environmental conditions that could potentially be used in assisted adaptation restoration activities. This knowledge can be used by conservation managers and ecosystem restoration practitioners to identify optimal donor sites for transplant material that may be tested for use in future‐proofing against current and future climate change scenarios. We combined genome‐wide SNP data and environmental variable data to (i) determine the most important variables exerting selective pressure; (ii) identify candidate genotypes associated with tested environmental variables, and (iii) develop guidelines to assist with source site selection for restoration in a habitat‐forming species.

## 2 Methods

### 2.1 Sample Collection

**FIGURE 2:**
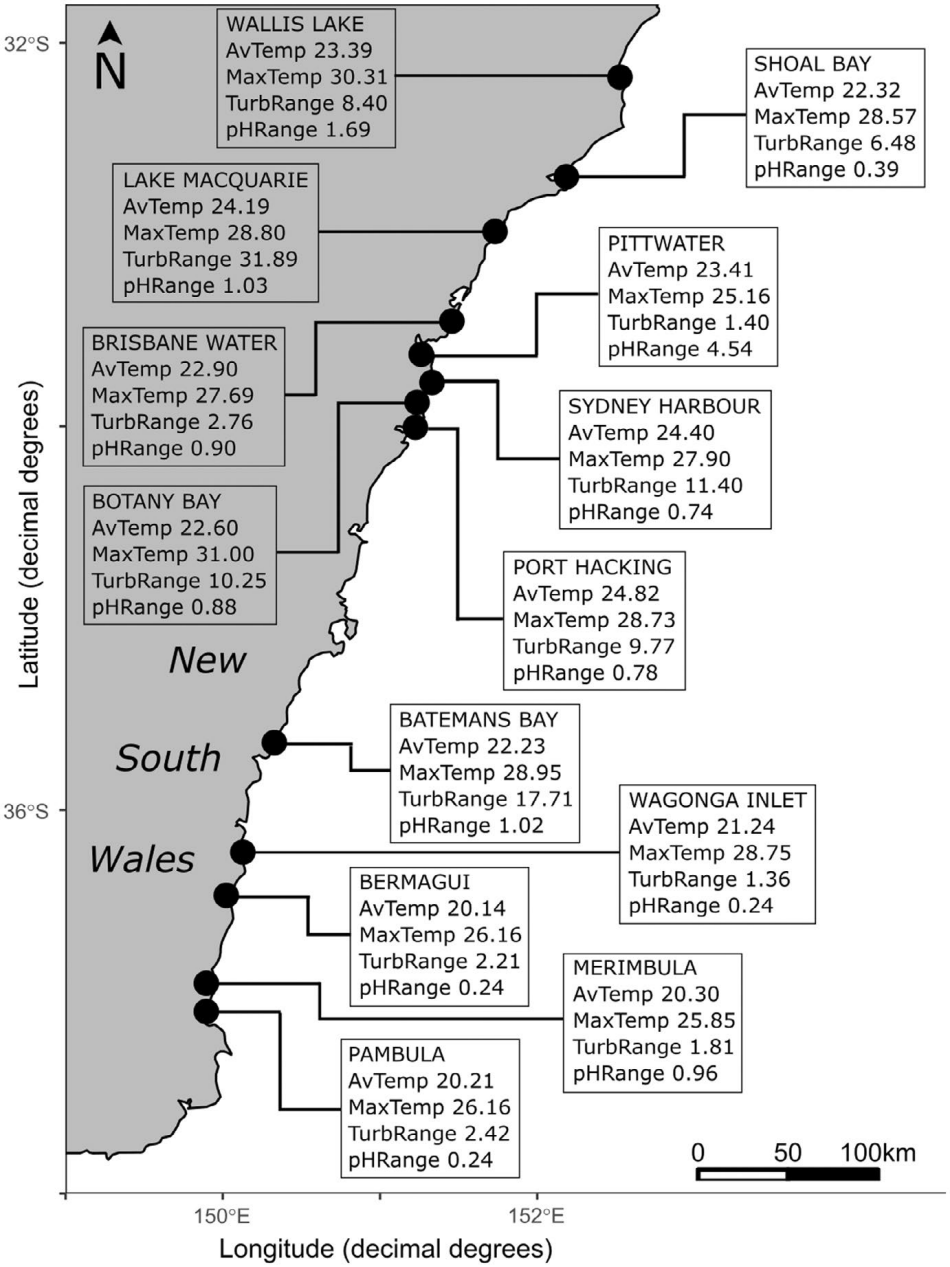
Map of locations of sampled *Posidonia australis* meadows in NSW. Data represent the four most important environmental variables for each meadow used in genotype × environment analyses. AvTemp = average temperature (°C); MaxTemp = maximum temperature (°C); pHRange = difference between highest and lowest observed pH values; TurbRange = difference between highest and lowest observed turbidity measure (NTU).

Thirteen estuaries were selected for sampling (Table 1) based on distribution maps (West and Glasby 2022), with one meadow within each estuary selected due to the availability of environmental data from that location. These meadows spanned the known range of *P. australis* in New South Wales (NSW), from Wallis Lake in the north to Pambula in the south (Figure 2). Samples were haphazardly collected in shallow water (1–1.5 m) at low tide using a snorkel or via wading in March–April 2022. *P. australis* shoots were hand collected by severing at the rhizome using secateurs. Samples were taken with a minimum distance of 2 m apart to reduce the likelihood of sampling clonal ramets. Thirty shoots were collected from each meadow, with 25 being the recommended minimum number of samples required to comprehensively sample a population genome using DaRT arrays (Edet et al. 2018; Mijangos et al. 2022; Sansaloni et al. 2011).

**TABLE 1:** Pairwise correlation analysis of twenty environmental variables initially assessed for use with GEAs: Average rainfall per year in mm (Av.Rain.yr), minimum rainfall per year (mm) (Min.Rain.yr), maximum rainfall per year (mm) (Max.Rain.yr), change in annual rainfall (mm) (Rain.change.yr1), average summer temperature in°C (AvTemp), maximum summer temperature (°C) (MaxTemp), summer temperature range (°C) (TempRange), maximum observed pH value (MaxpH), minimum observed pH value (MinpH), pH value range (pHRange), average salinity in practical salinity units (PSU) (AvSal), minimum observed salinity (PSU) (MinSal), maximum observed salinity (PSU) (MaxSal), salinity range (PSU) (SalRange), change in salinity values per year (PSU) (Salchange.yr1), average observed turbidity value in nephelometric turbidity units (NTU) (AvTurb), minimum observed turbidity value (NTU) (MinTurb), maximum observed turbidity value (NTU) (MaxTurb), change in turbidity values per year (NTU) (Turbchange.yr1) and turbidity range (NTU) (TurbRange).

	Av.Rain.yr	Max.Rain.yr	Min.Rain.yr	Rain.change.yr	AvTemp	MaxTemp	TempRange	MaxpH	MinpH	pHRange	AvSal	MaxSal	MinSal	SalRange	Salchange.yr	AvTurb	MaxTurb	MinTurb	TurbRange
Av.Rain.yr																			
Max.Rain.yr	**0.72**																		
Min.Rain.yr	**0.88**	**0.78**																	
Rain.change.yr	−0.13	−0.36	−0.36																
AvTemp	**0.82**	**0.79**	**0.82**	−0.33															
MaxTemp	0.19	0.17	0.07	−0.25	0.40														
TempRange	−0.30	−0.43	−0.42	0.09	−0.32	0.66													
MaxpH	0.42	0.39	0.43	0.11	0.30	−0.35	−0.52												
MinpH	0.04	−0.36	−0.05	0.34	−0.30	−0.38	0.06	0.43											
pHRange	0.44	0.52	0.48	0.03	0.40	−0.28	−0.58	**0.97**	0.20										
AvSal	−0.20	−0.13	0.05	−0.07	−0.15	−0.35	−0.12	0.52	0.55	0.42									
MaxSal	0.59	0.49	0.55	0.00	0.47	−0.29	−0.52	0.61	0.22	0.61	0.20								
MinSal	−0.45	−0.56	−0.47	0.02	−0.37	0.44	**0.75**	−0.27	0.26	−0.36	0.59	−0.50							
SalRange	−0.61	−0.55	−0.58	0.01	−0.49	0.35	0.61	−0.60	−0.15	−0.61	−0.49	0.64	**−0.99**						
Salchange.yr	0.19	−0.09	0.07	−0.23	0.34	0.55	0.33	−0.61	−0.26	−0.59	−0.17	0.24	−0.03	0.07					
AvTurb	−0.09	0.22	−0.17	−0.42	0.10	0.62	0.39	−0.37	**−0.75**	−0.20	−0.47	0.13	−0.43	0.41	0.15				
MaxTurb	−0.33	0.07	−0.16	−0.57	−0.20	0.23	0.28	−0.40	−0.59	−0.28	−0.24	0.02	−0.39	0.35	−0.02	**0.82**			
MinTurb	0.19	0.08	0.22	−0.10	0.52	0.48	0.13	−0.18	−0.42	−0.09	−0.08	0.15	0.10	−0.06	0.69	0.24	−0.01		
TurbRange	0.21	0.19	0.31	−0.38	0.34	0.24	−0.06	0.00	−0.23	0.07	0.07	−0.16	0.25	−0.25	0.15	0.16	0.17	−0.06	
Turbchange.yr	0.21	0.08	0.23	−0.06	0.54	0.46	0.11	−0.15	−0.38	−0.07	−0.06	0.15	0.13	−0.08	0.69	0.19	**1.00**	0.06	0.16

*Note:* Significant correlation values are indicated in bold.

Immediately after collection, tissue was removed from each sample by stripping leaf sheaths from the base of the shoot and rhizome and trimming out a 25 mg piece of meristem using a clean scalpel blade. These subsamples were then placed in 15.0 mL Corning centrifuge tubes containing 8 mL of hexadecyltrimethylammonium bromide (CTAB) lysis buffer (100 mM TrisHCl, 20 mM EDTA, CTAB 2% w/v, NaCl 1.5 M), 80 μL sodium dodecylsulfate, and 2 μL β‐mercaptoethanol.

### 2.2 DNA Extraction

Cell lysis was carried out by holding tissue subsamples at 65°C in an oven for 4 days. Tubes were vortexed every second day to rehomogenise the tube contents. The hot CTAB lysis buffer and tissue subsample were decanted into a sterilised mortar and milled until a homogenous paste formed. The mixture was returned to the 15 mL tube and another 2 μL of β‐mercaptoethanol was added. Tubes were vortexed and held at 65°C for another 2 days.

Samples were cooled to room temperature and purified by addition of an equal volume of 24:1 chloroform isoamyl. Tubes were centrifuged in a refrigerated centrifuge for 1 h at 4700 rpm at 4°C. After centrifugation, the aqueous phase was retained in a fresh 15 mL tube and DNA was precipitated by the addition of 0.66 volume of chilled 100% isopropanol. Tube contents were mixed by inversion. Samples were centrifuged at 4700 rpm for 1 h at 4°C. After centrifugation, the DNA formed a pellet, and the supernatant was disposed of. The DNA pellet was cleaned by the addition of 500 μL of 70% ethanol, mixed by inversion, and centrifuged at 4700 rpm for 30 min at 4°C. The supernatant was removed, and the DNA pellet was air‐dried to remove residual ethanol. The DNA was resuspended by the addition of 80 μL 0.1% TE buffer held overnight at room temperature. Extracted DNA was quality checked using NanoDrop, Qubit assay, and gel electrophoresis.

### 2.3 Single Nucleotide Polymorphism Genotyping

For SNP genotyping, 20 μL of extracted DNA (min concentration of 15 ng/μL) was sent to Diversity Arrays Technology Pty Ltd. (Canberra, Australia) (DArT). The DArT organisation provides a process pipeline of whole‐genome profiling without the need for a reference genome (Jaccoud et al. 2001). This uses DArTseq technology, which combines a proprietary genome complexity reduction method (Kilian et al. 2012) with next‐generation sequencing techniques, to detect single nucleotide polymorphisms (SNPs) and silicoDArT markers. As the method is proprietary, readers are directed to the DArT Pty Ltd. website (https://www.diversityarrays.com/technology‐and‐resources/dartseq/), and the processes discussed in Sansaloni et al. (2011) and Edet et al. (2018). Six technical replicates were included for subsequent sequencing error testing.

### 2.4 SNP Quality Control Filtering

In total, 342 individual *P. australis* were initially genotyped with the DArTseq platform, yielding a total of 11,382 SNP loci with a mean read depth of 7.07% and 20.73% missing data. Sequencing error was estimated by calculating the maximum proportion of allelic differences (bitwise distance) found between six pairs of technical replicates using *bitwise.dist* in the R package *poppr*, (Kamvar, Tabima, and Grunwald 2014), with 0.9% set as the error threshold. No sequencing errors above this threshold were detected so technical replicates were then removed from the dataset. To enhance the quality of SNPs and to optimise the number of loci available for identification of candidate SNPs under potential selection, a data filtering strategy was employed using several functions in the *R* package *dartR* v.2.7.2 (Gruber et al. 2019; Mijangos et al. 2022). Data were filtered by applying a locus call rate of 0.67 and an individual call rate of 0.25. A reproducibility threshold of 0.99 was applied, and read depth filter parameters were set at 2–50. SNPs were thinned by setting the MAF to default (0.01). After filtering, a total of 3277 SNP loci for 311 genotypes across the 13 populations were retained.

### 2.5 Management of Clones

Although long‐lived, slow‐growing seagrasses are capable of sexual reproduction and via the production of vegetative ramets, in those locations where little to no sexual reproductivity occurs, a substantial amount of genetic variation arises through somatic mutation within clonal ramets (Reusch, Baums, and Werner 2021; Wang et al. 2019; Yu et al. 2020). Expansive vegetative growth within meadows can lead to resampling the same clone. The inclusion of clonal replicates can violate basic assumptions of panmixia and Hardy–Weinberg equilibrium; therefore, standard population genetic analyses usually require some form of clone correction (Kamvar, Tabima, and Grunwald 2014). In contrast, methods that identify putatively adaptive SNPs rely on the presence of outliers, and for modular organisms or those that rarely reproduce sexually, clones are likely to contain most of the standing stock of adaptive SNPs due to somatic genetic variation (SoGV) (Wang et al. 2019; Yu et al. 2020; Reusch, Baums, and Werner 2021). Consequently, our outlier detection analyses were conducted on the complete dataset.

### 2.6 Genotype–Environment Associations

Environmental variables used in genotype–environment associations (GEAs) were sourced from multiple datasets and included precipitation, temperature, salinity, pH, and turbidity metrics. Daily catchment rainfall data from the last 20 years (2003–2023) was retrieved from weather stations managed by the Australian Government Bureau of Meteorology. Data for 20 estuarine environmental variables were retrieved and compiled from three different sources: (1) New South Wales Estuary Water Quality Data Compilation (EWQDC) generated by the Department of Planning, Industry, and Environment (State Government of NSW and NSW Department of Climate Change 2024); (2) the New South Wales Food Authority Shellfish Program (FASP); and, (3) the Transforming Australian Shellfish Production Project portal (TASPP; University of Technology Sydney et al. 2024). These datasets were selected as they provide the best available spatio‐temporal representation, despite having different spatial parameters, sampling frequencies and collection methods. The EWQDC provides data for 160 estuaries, including the 13 sampled in this study, and was used to analyse recent climate change effects (Scanes, Scanes, and Ross 2020). Sampling for EWQDC was mostly undertaken during warmer months (October–April), with salinity, pH and turbidity repeatedly measured at ~0.5 m depth within two or three sections of each estuary from a drifting vessel using a calibrated water‐quality sonde. EWQDC data were collected between 2007 and 2020. Sampling was focussed on one of three regions (northern, central, or southern) across the state every third year, with reduced sampling frequency in the other regions rather than annual sampling in each estuary. Similarly, the FASP data were spot measurements of temperature and salinity taken between 2004 and 2024 at depths of ~0.5 m. FASP sampling was designed to detect adverse estuarine conditions (to inform shellfish harvesting regimes) after significant rainfall events and then again when water quality had improved. In contrast, data from the TASPP were derived from continuous logging of water temperature and salinity in shellfish harvest areas by fixed sensors. Data from TASPP were only available for certain estuaries that support oyster aquaculture. Whilst the years of data collection varied across estuaries and there were gaps due to sensor replacement/maintenance, most had data recorded between 2021 and 2023, with some estuaries having data as early as 2016 and as late as 2024.

Environmental data for Batemans Bay, Botany Bay, Merimbula, Pambula, Port Stephens, and Wagonga Inlet were derived from the EWQDC, TASPP, and FASP, whereas data for Bermagui, Brisbane Water, and Wallis Lake were only available from EWQDC and FASP. Only data from EWQDC were available for the remaining four estuaries: Lake Macquarie, Pittwater, Sydney Harbour, and Port Hacking.

Each dataset was scrutinised so that metrics were derived from monitoring stations within or adjacent to *P. australis* meadows in each estuary. Measurements were generally taken ~1.5 km or less from *P. australis* (most TASPP and FASP sites were 100's of metres) from meadows; however, there were several estuaries where sites further away than this were included. These sites were mainly from the EWQDC sampling (but one TASPP sensor was ~2.5 km from *P. australis* ) and were retained to either ensure adequate replication or because samples were taken in the central sections of large estuaries where *P. australis* fringed the shoreline. A notable exception was for Batemans Bay, where limited data were available, so data were taken from locations in the lower reaches of the Clyde River and a small number of sites from the adjacent Cullendulla Creek. Additionally, data from the EWQDC for Botany Bay were sampled in the sections of the Georges River, but the TASPP and FASP temperature and salinity data came from within Botany Bay. Importantly, both these tidal rivers flow directly into their respective embayments and influence local environmental conditions. Data were then filtered to only warmer months based on the sampling periods in the EWQDC dataset (October–April) and limited to daylight hours to match sampling periodicity in the EWQDG and FASP datasets. Any spurious values or outliers were removed, and data were transformed into daily averages to calculate mean, minimum, and maximum values for each environmental variable. Monthly averages for each environmental variable were also calculated and used to determine the rate at which variables changed within each estuary per year from coefficients of generalised linear models fit with a gaussian distribution.

Data retrieval and treatment yielded a total of 20 environmental variables for an initial assessment with GEAs. They were average rainfall per year in mm (Av.Rain.yr), minimum rainfall per year (mm) (Min.Rain.yr), maximum rainfall per year (mm) (Max.Rain.yr), change in annual rainfall (mm) (Rain.change.yr^1^), average summer temperature in°C (AvTemp), maximum summer temperature (°C) (MaxTemp), summer temperature range (°C) (TempRange), maximum observed pH value (MaxpH), minimum observed pH value (MinpH), pH value range (pHRange), average salinity in practical salinity units (PSU) (AvSal), minimum observed salinity (PSU) (MinSal), maximum observed salinity (PSU) (MaxSal), salinity range (PSU) (SalRange), change in salinity values per year (PSU) (Salchange.yr^1^), average observed turbidity value in nephelometric turbidity units (NTU) (AvTurb), minimum observed turbidity value (NTU) (MinTurb), maximum observed turbidity value (NTU) (MaxTurb), change in turbidity values per year (NTU) (Turbchange.yr^1^) and turbidity range (NTU) (TurbRange). This dataset was first analysed using pairwise scatterplots to reduce dimensionality/collinearity using the function *pairs.panels* in the R package *LEA*. This was carried out as some GEAs are subject to problems (inflation of variance of regression parameters) when highly correlated predictors are used (Dormann et al. 2013). Generally, *r* > 0.7 is regarded as a good threshold for removing correlated predictors (Dormann et al. 2013) (Table 1). Collinearity was further checked using analysis of variance inflation factors (VIF) (Legendre and De Cáceres 2013) via the *vif.cca* function in the *R* package *vegan* (Oksanen et al. 2013). Variable reduction through tests of collinearity and variance inflation resulted in the retention of nine environmental variables for use in further analyses: AvTemp, MaxTemp, TempRange, MinpH, pHRange, AvSal, MaxSal, MinTurb, and TurbRange (Table 2).

**TABLE 2:** Sampling meadows for *Posidonia australis* in NSW with associated environmental predictor variables used in GEA and GF analyses.

Meadow location	Estuary type	Lat (°S)	Long (°E)	Samples sequenced (*n*)	AvTemp (°C)	MaxTemp (°C)	TempRange (°C)	pHRange	MinpH	MaxSal (PSU)	AvSal (PSU)	TurbRange (NTU)	MinTurb (NTU)
Wallis Lake	Lake	32.21	152.50	25	23.39	30.31	13.64	1.69	7.89	41.99	33.46	8.40	0.05
Port Stephens	River	32.71	152.17	25	22.32	28.57	13.57	0.39	7.88	38.96	30.64	6.48	1.10
Lake Macquarie	Lake	33.09	151.66	30	24.19	28.80	12.82	1.03	7.84	37.22	34.28	31.89	0.02
Brisbane Water	Lake	33.44	151.33	27	22.90	27.69	12.90	0.90	7.92	36.86	31.13	2.76	0.14
Pittwater	Lake	33.64	151.28	30	23.41	25.16	4.41	4.54	8.00	35.62	35.08	1.40	0.22
Sydney Harbour	River	33.80	151.27	30	24.40	27.90	6.62	0.74	7.21	25.44	29.77	11.40	0.87
Botany Bay	River	34.00	151.21	25	22.60	31.00	18.03	0.88	7.3	42.70	31.60	10.25	2.34
Port Hacking	River	34.05	151.14	25	24.82	28.73	7.93	0.78	7.52	36.04	28.44	9.77	0.33
Batemans Bay	River	35.73	150.20	30	22.23	28.95	12.45	1.02	7.26	38.84	27.77	17.71	0.70
Wagonga Inlet	Lake	36.22	150.12	30	21.24	28.75	15.75	0.24	8.04	42.51	31.71	1.36	0.11
Bermagui	River	36.42	150.06	30	20.14	26.16	11.66	0.24	7.67	37.10	34.66	2.21	1.28
Merimbula	Lake	36.89	149.91	25	20.30	25.85	11.72	0.96	8.06	40.59	30.95	1.81	0.30
Pambula	River	36.94	149.90	30	20.21	26.13	11.45	0.19	7.90	42.38	32.40	2.42	0.55

*Note:* Headers = latitude (Lat), longitude (Lon), average summer temperature in°C (AvTemp), maximum summer temperature (°C) (MaxTemp), summer temperature range (°C) (TempRange), minimum observed pH value (MinpH), pH value range (pHRange), average salinity in practical salinity units (PSU) (AvSal), maximum observed salinity (PSU) (MaxSal), minimum observed turbidity value (NTU), and turbidity range (NTU) (TurbRange). Environmental values specific to sampling sites as derived from available datasets and not representative of the entire estuary. Estuary types derived from Scanes, Scanes, and Ross (2020).

### 2.7 Genome‐Wide Signatures of Adaptive Variation

Genomic scans for adaptive divergence were carried out to identify candidate SNPs potentially under selective pressure using three different models: Redundancy analysis (RDA) (Legendre and Legendre 2012), Principal Component Analysis for Outlier Detection (PCAdapt) (Luu, Bazin, and Blum 2017) and Latent Factor Mixed Models (LFMM2) (Frichot et al. 2013). Redundancy analysis generates a constrained ordination that models linear relationships between genomic variance and environmental predictor variables (Legendre and Legendre 2012). This way, covarying allele frequencies that are associated with multiple environmental metrics can be identified. Here, tails of distributions calculated as part of the GEA were analysed for outliers with a 2.5 standard deviation cutoff (two‐tailed *p*‐value = 0.0005). Principal component analysis for outlier detection (PCAdapt) scans for outlier SNPs based on population structure through principal component analysis (PCA) (Privé et al. 2020). Five principal components (*K* = 5) were found to capture most of the background genetic variation based on analysis of ADMIXTURE and PCA. Those outlier SNPs that deviated significantly from neutral background structure based on principal components (Bonferroni correction with adjusted *p*‐values < 0.05) were regarded as candidate SNPs, putatively under selective pressure. LFMM2 tests for linear relationships between genetic variants and environmental predictors with random latent factors using the least‐squares method.

To infer population structure, individual ancestral coefficients were estimated based on a sparse non‐negative matrix factorisation (SNMF) method. This was implemented using the *snmf* function in the *R* package *LEA* v3.10.2 (Frichot and François 2015). Ancestry coefficients were determined for 1–13 ancestral populations (*K*) by generating an entropy criterion that evaluates the fit of the statistical model to the data using a cross‐validation technique (Frichot and François 2015). From 100 repetitions, the *K* value with the lowest cross‐entropy score was selected. Next, the optimal factor, *K* = 8, was used to inform the LFMM to identify whether allele frequencies were correlated with any of the environmental variables. Statistical power of associations was increased by imputing missing genotype data via the *gl.impute* function in the *dartR* package using the *nearest neighbour* option. Subsequently, the function *lfmm_ridge* was used to compute a regularised least‐squares estimate using a ridge penalty. Individual associations between each SNP frequency and each environmental variable were assessed using statistics test calibrated using genomic inflation factor (function *lfmm_test*). Corrections for multiple comparisons were applied with the Benjamini‐Hochberg algorithm with a false discovery rate (FDR) threshold of 5% (Benjamini and Hochberg 1995). Significance associations was determined using a threshold of 0.001 as the probability of finding a false positive result increases with lower thresholds (Ahrens, Byrne, and Rymer 2019). Candidate SNP loci were retained for downstream analysis when they were identified by at least two out of the three methods.

The Gradient Forest (GF) algorithm was then used to describe the associations of environmental and genetic variables (Ellis, Smith, and Pitcher 2012). The turnover functions in GF allow for inference of the environmental predictors driving observed changes in allele frequency (Fitzpatrick and Keller 2015). The GF analysis was run in the *R* package *gradientForest* (Ellis, Smith, and Pitcher 2012), using a regression tree‐based approach to fit a model of responses between genomic data and environmental variables (Capblancq et al. 2020). Turnover in adaptive genetic variation were modelled on the predictor variables using the candidate SNPs, identified via GEAs, as the response variables. The machine learning algorithm partitioned allele frequencies at numerous split values along each environmental gradient and calculated the change in allele frequencies for each split. The split importance (i.e., the amount of genomic variation explained by each split value) was cumulatively summed along the environmental gradient and aggregated across alleles to build a non‐linear turnover function to identify loci that were significantly influenced by the predictor variable (Ellis, Smith, and Pitcher 2012). The analysis was run over 500 regression trees for each of the nine environmental predictor variables with all other parameters at default settings.

### 2.8 Data Limitations and Interpretation

It is important to note that methods used to explore candidate SNPs under putative selection were based on a simplistic approach. Reference alleles were deemed to represent the neutral or ancestral allelic state, thus any variant allele was regarded as a derived state. Given that identified candidate SNPs are significantly correlated with their relative environmental predictor it can be assumed that the SNPs are likely to have a functional purpose (Brookes 1999). Furthermore, it can be assumed that, in response to selective pressures exerted by environmental predictors, there will be changes in allele frequency amongst candidate SNPs and that those pressures may maintain any variant alleles (Orgogozo, Morizot, and Martin 2015). Additionally, heterozygotic SNPs may be maintained due to overdominance (heterozygotic advantage) or balancing selection (Hedrick 2012), and although alternate homozygotes may be adaptive, the function of the specific SNP can help to explain the variant response.

## 3 Results

### 3.1 Genome‐Wide Signatures of Adaptive Differentiation

Redundancy analysis and LFMM each identified candidate SNP loci exhibiting significant genotype–environment associations for each of the nine environmental predictors tested. PCAdapt detected 141 outlier SNPs with significant correlations (*q*‐values < 0.1, with FDR bounded by 0.1). LFMM detected 1026 outlier SNPs that were significantly correlated with each of the environmental predictors and RDA detected 514. Permutational analysis of variance (PERMANOVA) revealed the RDA model to be highly significant overall (*p* = 0.001), and nine constrained axes were also highly significant (*p* ≤ 0.002). There were no common candidate SNP loci detected by all three methods. However, 141 loci were detected by at least two methods, LFMM and PCAdapt. The absence of concordance amongst all three methods is likely given that GEA algorithms have varying sensitivities to detecting loci under selection, use different methods for controlling for demography, and adopt different association algorithms. The 141 significantly associated candidate loci identified by LFMM and PCAdapt were then used in the subsequent GF analysis (Table 3).

**TABLE 3:** Numbers of outlier SNPs detected for each environmental predictor using two GEA algorithms: redundancy analysis (RDA) and latent factor mixed models (LFMM2).

Variable	AvTemp	TurbRange	MaxTemp	pHRange	MinpH	TempRange	MinTurb	AvSal	MaxSal
RDA	80	80	94	84	74	44	110	150	88
LFMM2	662	157	110	317	126	239	184	100	213

**FIGURE 3:**
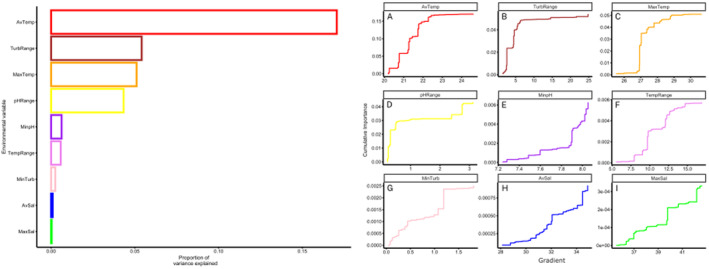
*Gradient forest* analysis for 141 candidate SNPs in *Posidonia australis* meadows. *Left*—relative importance for each environmental variable that describes frequencies in allelic turnover. *Right*—cumulative importance curves showing overall pattern of genomic compositional change (*y*‐axis) for nine environmental variables (*x*‐axis). Turnover functions for each curve are aggregated across all candidate loci. The maximum height of each curve indicates the relative amount of allelic turnover, and the relative importance of each environmental variable.

Gradient forest analyses confirmed all candidate SNP loci to be significantly correlated with environmental data (*R* ^2^ values > 0; mean = 0.06, range 0.0001–0.30). Overall, AvTemp, TurbRange, MaxTemp and pHRange were found to be the most important predictors of adaptive genomic variation, explaining ~17%, 6%, 5.5% and 5% of variation, respectively (Figure 3 left). Conversely, AvSal and MaxSal had the weakest effect. Temperature‐associated environmental predictors accounted for the greatest proportion (~23%) of genomic variance among candidate SNPs. Plots of turnover functions from the GF model show weighted cumulative importance values and variable allelic turnover slopes along each environmental predictor gradients (Figure 3 right). The most important predictor, AvTemp (Figure 3A.), had a steep gradient with a series of abrupt gradient changes at ~20.6°C, ~21.2°C and ~22°C, with very minor slope beyond 22.5°C. A similar slope and pattern of abrupt turnover occurred in TurbRange (Figure 3B) with a large change between ~2 and 5 NTU. For MaxTemp (Figure 3C), a large turnover in allelic composition occurred at ~26.9°C, followed by small steps between 27°C and 29°C where the slope flattened and thereafter there was little genomic change. Allelic turnover for pHRange (Figure 3D) followed a very steep slope with large turnover between 0 and 0.5, followed by a smaller change at 2.5 and again at ~2.7. There was a pattern of gradual change in MinpH (Figure 3E) with a concave slope until pH reached ~7.9, where there was an abrupt change with the slope rising steeply until pH 8.0. TempRange (Figure 3F) had a gradual step‐wise slope until 7.5°C, followed by an abrupt change at ~10°C and another at ~12°C.

MinTurb (Figure 3G) followed a similar pattern of gradual change until an abrupt change at ~1.2 NTU, followed by a flattened slope and then another jump at ~2 NTU. Slopes in the salinity associated turnover plots (Figure 3H,I) were somewhat concave with a consistent turnover amongst the constituent outlier SNPs with minor abrupt changes at ~32 PSU and again at ~35 PSU in AvSal and at ~40 PSU and ~42.5 in MaxSal.

### 3.2 Outlier SNP Gene Function

To explore the biological function of candidate SNPs for the four most important environmental variables (AvTemp, TurbRange, MaxTemp and pHRange), sequences were compared to the global GenBank database (Clark et al. 2016) using the BLASTn algorithm (Johnson et al. 2008) available via the NCBI webportal. This process searches for and offers a percentage match score for similar gene sequences stored in Genbank that have been annotated with an identified physiological function. Of the sequences tested, three returned a confident (> 95%) match with an annotated gene (Clark et al. 2016). The AvTemp associated candidate SNP, locus ID 101924221‐25‐G/C sequence TGCAGGTCAATTGTTATTCATTGAATTCTGTTGGCCCTACAGTGGAGACGTTCAGTGGACCAAAAAGAT, matched with Genbank LOC117926718 derived from the riverbank grape, *Vitis riparia* . Gene function is associated with the chloroplastic rhomboid‐like protein 10 located in organelle membranes. According to Huntley et al. (2015) it functions as

Catalysis of the hydrolysis of internal alpha‐peptide bonds in a polypeptide chain by a catalytic mechanism that involves a catalytic triad consisting of a serine nucleophile that is activated by a proton relay involving an acidic residue (e.g. aspartate or glutamate) and a basic residue (usually histidine).

Although the primary function of chloroplastic rhomboid‐like protein 10 is to cut and regulate other proteins, this activity may affect cellular response to temperature stress by maintaining protein quality and function (elevated temperatures may damage protein folds) within chloroplasts (Urban 2006).

The two other matched SNP sequences were associated with the TurbRange variable: locus ID 101761005‐56‐T/C, sequence: TGCAGGACAAGATGTTGAAGTTGGGTGTTTTGCCGAATGCCCATACATGCAAGTCGTTGATCTTTGGCA matched with LOC108511391 from the date palm, *Phoenix dactylifera* and the gene codes for a pentatricopeptide repeat‐containing protein At5g40400 (Clark et al. 2016). Its function is associated with protein binding and its action affects function and biogenesis of cell organelles which, in turn, drives photosynthetic, respiratory, developmental functions and environmental responses (Barkan and Small 2014). Locus 101924221‐25‐T/C, sequence TGCAGGTCAATTGTTATTCATTGAACTCTGTTGGCCCTACAGTGGAGACGTTCAGTGGACCAAAAAGAT matched with LOC107925473 from cotton, *Gossypium hirsutum* (Clark et al. 2016) which is a protein SSUH2 homologue. Located within cell membranes, this molecule enables serine‐type endopeptidase activity which allows enzymes to cleave peptide bonds in proteins (Page and Di Cera 2008). Although not specifically associated with their individual environmental variables per se these last two SNPs are associated cellular function.

### 3.3 Distribution of Allelic Diversity Among Adaptive SNPs in Response to Environment

**FIGURE 4:**
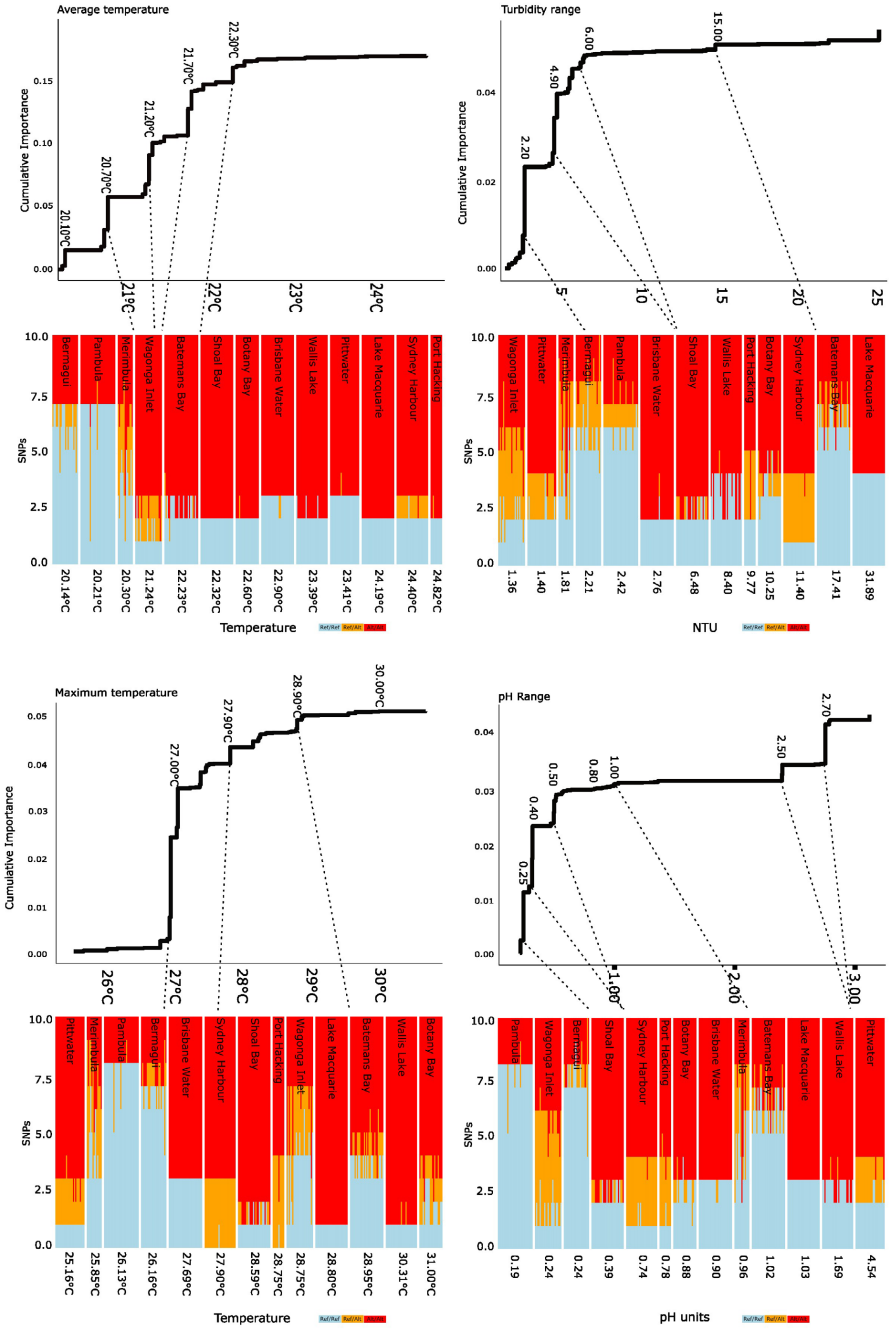
Site based SNP allele distributions mapped to allelic turnover plots and environmental gradients for the four most important variables: average temperature, turbidity range, maximum temperature, and pH range. Reference homozygote alleles are light blue, heterozygotic loci are orange, and alternate homozygotes are red. Dotted lines connect approximate point of allelic turnover between turnover plot and allelic distribution histograms.

There was no latitudinal pattern of allelic turnover, reflecting the unique and highly variable environmental conditions within each estuary. For the four most important environmental predictors (which collectively account for ~30% of variation) the proportionate distribution of alleles among individuals, aggregated into meadows, was mapped using stacked histograms. These were then cast against the slope of allelic turnover along each environmental gradient generated by GF to visualise patterns of adaptive response (Figure 4). Here, reference homozygote alleles are shown in light blue, heterozygotic loci as orange, and alternate homozygotes as red.

For AvTemp, two southern meadows with average temperatures below 20.21°C (Pambula and Bermagui) have retained the reference allele across most candidate SNPs. Heterozygotes were mostly found in meadows with average temperatures between 20°C and 21°C with some reference homozygotes retained in Sydney Harbour. Alternate homozygotes were found for most candidate SNPs in meadows where average temperatures were > 22°C (meadows north of and including Wagonga Inlet). Candidate SNPs associated with TurbRange underwent the majority of allelic turnover where range was > 2 but < 6 NTUs and, with the exception of Batemans Bay, where TurbRange was between 1.36 and 2.42 NTU tended to exhibit greater proportions of reference homozygotes than those > 2.5 NTUs.

The greatest allelic change occurred for MaxTemp at ~27°C. With the exception of Pittwater, all estuaries with MaxTemp < 27°C exhibited a greater proportion of reference homozygotes than those with MaxTemp range 27.7°C–28.8°C which tended to have higher proportions of heterozygotes. A higher proportion of alternate homozygotes was observed when MaxTemp > 28.8°C. Patterns associated with pHRange indicated that those meadows with a range between 0.19 and 0.24 retained reference homozygotes, and where pHRange was > 0.39 there were larger proportions of alternate homozygotes. Heterozygosity was scattered amongst the meadows but was dominant among candidate SNPs at Wagonga Inlet.

## 4 Discussion

### 4.1 Signatures of Adaptation

The identification of donor seagrass meadows that are genetically pre‐adapted to likely future climate conditions has been suggested to be a critical part of targeted efforts to future‐proof seagrass habitats (Tan et al. 2020; Pazzaglia et al. 2021b). In the present study, ~17% of SNPs were identified as candidates under selection and 141 (0.04%) were significantly correlated with important environmental variables in estuaries that are predicted to be altered under climate change. Genotypes that were potentially pre‐adapted to specific environmental conditions can provide a genomic foundation for use in future restoration planning that aims to boost climate‐resilience. Correlations suggest that *P. australis* has, at least partly, adapted to contemporary abiotic conditions at the genomic level among estuaries that have undergone environmental change over the last two decades in NSW (Scanes, Scanes, and Ross 2020), and modification since European settlement. Studies using SNPs in other seagrass taxa have found similar signatures of adaptation. For example, *Nanozostera capensis* (Setchell) Tominson & Posluszny, 2001 meadows in South Africa, indicated a clear selective response to local temperature and rainfall regimes (Phair 2016; Phair et al. 2019). Similarly, Mediterranean meadows of *Posidonia oceanica* (Linneaus) Delile, 1813 exposed to extreme conditions had adaptive SNP loci linked to high temperatures and considerable reduction in genotypic diversity compared to meadows with more moderate thermal conditions (Nguyen et al. 2023). Together, these studies suggest that local adaptation through selection to extant environmental conditions can be used as a basis to design restoration strategies that aim to future‐proof seagrass meadows.

Habitats in which seagrasses typically reside are warming an order of magnitude faster in south‐eastern Australia than global models predict (Scanes, Scanes, and Ross 2020) highlighting the need for restoration to consider future temperature in provenance decisions. Temperature was the main driver of putative selection in this study and is a strong force causing selection and adaptation in many other marine species (e.g., Vranken et al. 2021; Cayuela et al. 2021). Indeed, three of the six most important environmental predictors (AvTemp, MaxTemp and TempRange, respectively) in our study were associated with temperature, and all generated very steep convex curves in allelic turnover with clear tipping‐points (Figure 3). Average temperature was also highly correlated with rainfall, which can affect estuarine salinity and turbidity. A selective response to higher average (> 22°C) and higher maximum (> 26.9°C) temperatures was apparent through the absence of heterozygotes and predominant fixation of alleles as alternate homozygotes in estuaries above these thresholds, suggestive of selection. For example, the temperature‐related SNP (locus ID 101924221‐25‐G/C) exhibited a steep change in allelic turnover at ~22.5°C, which is suggestive of a genomic response to generate enzymes that manage or remove chloroplastic proteins that may have been damaged (misfolded) by warming (Urban 2006). Meadows in the central and southern parts of the sampling range (Pittwater to Pambula) that experience lower average and maximum temperatures exhibited higher proportions of heterozygotes suggesting a lack of strong selective forces in these estuaries for loci under selection in other locations. Given that *P. australis* is endemic to cool, temperate waters (Short et al. 2007), meadows situated towards the northern (warmer) limit of its distribution in NSW are expected to exhibit this pattern (i.e., show signatures of selection as fixation), as they are potentially near their ceiling of thermal tolerance. The optimal temperature for *P. australis* in NSW estuaries is unknown, however, thermal optima (27.4°C) and the thermal maximum (36.7°C) were measured for *P. australis* in waters near Perth (Western Australia), at a similar latitude to the northernmost *P. australis* meadows in NSW at Wallis Lake (Said et al. 2024). Thermal optima vary with latitude, but a similar range (25°C–30°C) was obtained for two other persistent temperate species along the Western Australian coastline, *Posidonia sinuosa* and *Amphibolis antarctica* (Said et al. 2024). Further research is required to understand how the thermal limits of seagrasses might be influenced by indirect effects related to sediment processes (Marbà and Duarte 2010) such as sediment composition and/or the subsurface microbiome (Fuggle, Gribben, and Marzinelli 2023; Jongen et al. 2024; Walker et al. 2024), in which case these variables could be important to consider in the context of temperature changes.

Turbidity is predicted to change in estuaries as climate change alters rainfall patterns and runoff (Gillanders et al. 2011) which will have strong structuring forces for photosynthetic organisms in estuarine systems (Amri et al. 2021). The presence of an abrupt turnover of alleles associated with turbidity was found between 2 and 5 NTU concomitant increase in alternate homozygotes SNPs across the metropolitan estuaries (Sydney Harbour, Botany Bay and Port Hacking) as well as Batemans Bay and Lake Macquarie, and to a lesser extent Wallis Lake and Port Stephens., However, for the metropolitan estuaries two or three SNPs associated with TurbRange remain heterozygotic. An abrupt allelic turnover at ~5 NTU was observed in the twoSNP loci with identified metabolic gene functions Those estuaries with a low turbidity range (< 6 NTU: Brisbane Water, Pittwater, Wagonga Inlet, Bermagui, Merimbula and Pambula) generally retained a higher proportion of reference alleles. Patterns of allelic distribution at Batemans Bay and Wagonga Inlet, however, were contrary to the above. Batemans Bay retained reference homozygotes for two to three SNPs, even though it experiences a TurbRange of 17.71 NTU, whilst the low TurbRange meadow at Wagonga Inlet (1.36 NTU) exhibited heterozygosity in two outlier SNPs. These anomalous patterns indicate that there may be multiple factors associated with a genomic response in some loci, such as the presence of reproduction based genetic recombination (e.g., Ruan et al. 2021) or the presence of linked SNPs or multi‐SNP locus associations (Ehret et al. 2012). In a recent study of adaptive response of *Zostera marina* genotypes across two adjacent estuaries with nearly identical environmental gradients, Schiebelhut et al. (2023) suggest that it is highly likely that multiple mutational genetic pathways have led to the same phenotypic expression. They found that parallel shifts in allele frequencies across two locations, following the same environmental gradient, may not be detected by independent genome scans. This suggests that although populations may be subject to a similar pattern of selective pressure, the genomic response is of a continuous nature rather than binary. As a result, there may well be complex evolutionary predictability for polymorphic traits. The very high TurbRange for Batemans Bay was likely due to the effect of a single, short‐term pulse of highly turbid water (a flood event) that over‐inflated the turbidity range metric but generated little to no long‐term selective pressure on the seagrass genome. Indeed, even though the Batemans Bay meadow sits at the seaward margin of a drowned river valley, conditions are predominantly oceanic, with a low long‐term average turbidity of 4.08 NTU.

Sea water pH plays an important role in seagrass photosynthesis, where increased pH can result in a linear reduction in photosynthetic rate (Invers, Romero, and Pérez 1997). This biological response must be considered as estuaries can become acidic with ongoing climate change. Indeed, NSW estuaries have exhibited a 0.09 pH unit reduction per year since 2007 (Scanes, Scanes, and Ross 2020). The GF response curve for outlier SNPs associated with pHRange exhibited strong allelic turnover where the range spans ~0.2 to ~0.5 pH units, and only three estuaries exhibited a pH range < 0.3: Wagonga Inlet (0.24), Bermagui (0.24) and Pambula (0.19). Two of these southern meadows retained the reference homozygote (Bermagui and Pambula) across most SNPs, whereas Wagonga Inlet exhibited heterozygotes in most SNPs (following a similar pattern as TurbRange). The acquisition of alternate homozygotes for most pHRange associated SNPs across the central northern estuaries indicate putative adaptation to greater pH range in these meadows. The remaining genotype–environment associations presented here (MinpH, TempRange, MinTurb, AvSal and MaxSal) accounted for < 5% of genomic variation and are likely of little consequence for the understanding and informed selection of pre‐adapted donor meadows for future restoration in this region.

### 4.2 Time, Environmental Change, and Genomic Response

Adaptation via DNA polymorphism for most species generally occurs too slowly to keep pace with current climate change (Quintero and Wiens 2013), although some species are capable of rapid evolution at the genomic level in response to anthropogenically‐driven environmental change over the last 100–140 years (Hoffmann and Sgrò 2011). The detection of a selective response to current environmental conditions within the genome of *P. australis* in NSW is indicative of selection of existing genomic variation over long periods of time (thousands of years) (Bürger and Lynch 1995). It is likely that *P. australis* meadows comprise individual plants that have become isolated from what may have been more contiguous (or more connected) meadows along the NSW coast after the last glacial maximum (c. 12,000 years ago) (Sinclair et al. 2023). At this time, *P. australis* was likely to have had an equatorward range limit as far north as K'gari (Fraser Island), Queensland (~25° S) (Sinclair et al. 2023) and a greater genomic pool from which to adapt to southward shifting isotherms as the planet warmed (Eckert, Samis, and Lougheed 2008). Rising sea levels would have created new shallow‐coastal habitat as river valleys and embayments were flooded, resulting in more fragmented meadows (Williams et al. 2018). Selective pressures over this time are likely to have increasingly favoured individual genotypes that persist today. It is probable that meadows south of Wagonga Inlet, with greater proportions of reference homozygotes across most of the environmental variable gradients, retained most of their ancestral alleles in response to cooler water temperatures, greater water clarity and narrower pH range. Conversely, meadows where environmental conditions have resulted in the gradual change of alternate homozygotes (i.e., most meadows north of Batemans Bay) for outlier SNPs associated with rapidly changing variables (warming, acidification and increasing turbidity), means that whilst these populations are potentially adapted to conditions, they may also be at increased risk should these variables continue on their present trajectories. Furthermore, the retention of heterozygosity for some SNPs means that a level of adaptive capacity has been maintained within the genome to combat future change. The recent discovery of SoGV and non‐genetic components (e.g., transposable elements) in seagrasses could facilitate epigenetically regulated phenotypic plasticity in response to extreme stress events (Yu et al. 2020; Reusch, Baums, and Werner 2021), a particularly important element for this long‐lived genus of seagrasses (Arnaud‐Haond et al. 2012; Edgeloe et al. 2022).

This study highlights the need for improved estuarine monitoring of environmental variables and sampling of genetic variability to capture the range of highly variable habitats and better understand selection within estuaries. Here, environmental data were recorded at single geographic point/s within each estuary (that match the location of genetic samples) and should not be taken as representative of conditions across an entire estuary. In some cases, there was some distance between the location where environmental data was collected and the corresponding *P. australis* meadow (i.e., Batemans Bay [~7 km] and Botany Bay [~6 km]) which could cause a mismatch in the magnitude of environmental variation those meadows experienced. Most environmental data were recorded from the upper water column (< 0.5 m), and seagrass was sampled ~1–1.5 m deep however, many *P. australis* meadows in NSW are found at greater depths of 3–10 m (Larkum 1976). Moreover, a large proportion of the environmental data were collected only during warmer months, which likely missed peak rainfall events (which affect salinity and turbidity), and not all estuaries were regularly sampled on an annual basis over the 10‐year period. These limitations highlight a pressing need for comprehensive monitoring of environmental conditions across NSW estuaries and how these might affect seagrass persistence and adaptation. This need is particularly important given that southeast Australia is a climate change hotspot (Hobday and Pecl 2014) already experiencing extreme heatwaves, likely close to the upper limits of thermal maxima leading to changes in reproductive biology for taxa (e.g., Holland et al. 2024). Such monitoring is essential to determine the impacts of long‐term climate change patterns and extreme climate events (e.g., heatwaves, floods), which are projected to increase in frequency and intensity under future climate scenarios (Dey et al. 2019; Oliver et al. 2019). Given the ecological consequences of environmental change on foundation species (Wernberg et al. 2024), enhanced monitoring regimes that collect data relevant to species within estuaries (e.g., sampling at depths appropriate to seagrass meadows) would substantially improve research capacity and aid the development of mitigation strategies such as climate‐adjusted provenancing. Moreover, more detailed genetic sampling within each estuary at sites that experience different environmental conditions could help disentangle large scale evolutionary patterns and dispersal events from adaptation and selection. This could also be important for restoration as better adapted genotypes could be found within the same estuary and avoid issues associated with translocation among distant estuaries.

### 4.3 *Posidonia australis* Genomic Provenance, the First Step to a Donor Registry

We propose that the results of the present study can be used as a foundation for a ‘donor registry’ for matching existing environmentally pre‐adapted genotypes with projected climate conditions for meadow restoration. Tools exist for terrestrial plants (Rossetto et al. 2019) and marine species (Wood et al. 2024), however, there are no such resources for species inhabiting highly variable estuarine systems in which genomic patterns and climatic change are more difficult to predict. We provide here an example of how to identify which pre‐adapted genotypes to select for future‐proofing restoration programs, given a particular climate projection, using a simplistic calculation of potential future conditions (Table 4). Here, a 12‐year annual average change in temperature estimated for NSW estuaries by Scanes, Scanes, and Ross (2020) was used to generate an estimate of future temperatures on a decadal basis. Contemporary temperature measures reported in the present study were used as a base to which annual increases of 0.2°C year^−1^ were added. The results do not account for any confounding or mitigating factors and assume a constant rate of change. The calculation was applied to AvTemp and MaxTemp values, but predictions were not made for TurbRange or pHRange as a temporal history of changes in turbidity were not available and, although pH is anticipated to continue to reduce (Scanes, Scanes, and Ross 2020), the magnitude and trajectory of change is likely to apply to both maximum and minimum pH values resulting in a null or minimal effect on range values.

**TABLE 4:** Example of the potential application of genomic data to identify donor meadows to match future environmental conditions. Columns are shaded to improve readability.

Meadow location	Average temperature future predictions (°C)					Potential donor meadows			Maximum temperature future predictions (°C)					Potential donor meadows		
	Current	Change year^−1^	2030	2040	2050	2030	2040	2050	Current	Change year^−1^	2030	2040	2050	2030	2040	2050
Wallis Lake	23.39	+0.2	24.59	26.59	28.59	Port Hacking	NA	NA	30.31	+0.2	31.51	33.51	35.51	NA	NA	NA
Port Stephens	22.32	+0.2	23.52	25.52	27.52	Wallis Lake	NA	NA	28.57	+0.2	29.77	31.77	33.77	Wallis Lake	NA	NA
Lake Macquarie	24.19	+0.2	25.39	27.39	29.39	NA	NA	NA	28.8	+0.2	30.00	32.00	34.00	Wallis Lake	NA	NA
Brisbane Water	22.90	+0.2	24.10	26.10	28.10	Lake Macquarie	NA	NA	27.69	+0.2	28.89	30.89	32.89	Batemans Bay	Botany Bay	NA
Pittwater	23.41	+0.2	24.61	26.61	28.61	Port Hacking	NA	NA	25.16	+0.2	26.36	28.36	30.36	Brisbane Water	Port Stephens	Botany Bay
Sydney Harbour	24.40	+0.2	25.60	27.60	29.60	NA	NA	NA	27.90	+0.2	29.10	31.10	33.10	Wallis Lake	Botany Bay	NA
Botany Bay	22.60	+0.2	23.80	25.80	27.80	Lake Macquarie	NA	NA	31.00	+0.2	32.20	34.20	36.20	NA	NA	NA
Port Hacking	24.82	+0.2	26.02	28.02	30.02	NA	NA	NA	28.73	+0.2	29.93	31.93	33.93	Wallis Lake	NA	NA
Batemans Bay	22.23	+0.2	23.43	25.43	27.43	Pittwater	NA	NA	28.95	+0.2	30.15	32.15	34.15	Wallis Lake	NA	NA
Wagonga Inlet	21.24	+0.2	22.44	24.44	26.44	Botany Bay	Sydney Harbour	NA	28.75	+0.2	29.95	31.95	33.95	Wallis Lake	NA	NA
Bermagui	20.14	+0.2	21.34	23.34	25.34	Batemans Bay	Pittwater	NA	26.16	+0.2	27.36	29.36	31.36	Brisbane Water	Wallis Lake	Botany Bay
Merimbula	20.30	+0.2	21.50	23.50	25.50	Batemans Bay	Pittwater	NA	25.85	+0.2	27.05	29.05	31.05	Brisbane Water	Wallis Lake	Botany Bay
Pambula	20.21	+0.2	21.41	23.41	25.41	Batemans Bay	Pittwater	NA	26.13	+0.2	27.33	29.33	31.33	Brisbane Water	Wallis Lake	Botany Bay

*Note:* Data are predicted change in average and maximum temperatures based on multiplication of average annual change, as reported by Scanes, Scanes, and Ross (2020) for each of the seagrass meadows studied. Predictions for each decade are based on the addition of the calculated annual average change over the requisite number of years from the current data as at 2024. Potential donor meadows are those meadows with candidate SNPs showing contemporary adaptation ≥ the projected condition for the relevant scenario. NA = pre‐adapted donor material is not available. For example, to select a donor source for use in Port Hacking that is pre‐adapted to a maximum temperature predicted for 2040, readers select the line for Port Hacking in the left column and trace to the right to get the value for maximum temperature for 2040 (31.93°C) and continue to the right to find Wallis Lake under the Potential Donor Meadow column.

We note that correlation does not equate to causation and it will be important to experimentally demonstrate that the seagrass genotypes suggested to be pre‐adapted to specific environmental conditions here, do indeed perform as one might expect under projected environmental conditions. This should be a prerequisite to using assisted adaptation in any setting. Indeed, the information provided here could be considered part of a knowledge tool‐kit and used as a basis for manipulative experiments that physically test hypotheses about temperature tolerance among pre‐adapted genotypes. For example, plants from meadows identified as candidate donors ought to be physically subjected to future‐projected temperatures via mesocosm experiments under controlled conditions to identify upper limits or extended temporal tolerance to higher average temperatures. It would also be prudent to test whether thermally pre‐adapted genotypes also exhibit resilience (or maladaptation) to other potentially co‐occurring stressors, such as hyposalinity, light availability, or the effects of burial. Other important physical traits also need to be explored to determine whether heat‐adapted genotypes generate phenotypes that render them suitable for use in restoration programs. For example, plants that exhibit slow root growth will not readily anchor into sediment (which is crucial for restoration success), or plants may flower less frequently (or never), diminishing any long‐term population viability through reduced gene flow. Finally, it has been proposed that macrophyte restoration should employ a portfolio approach (Wood et al. 2019) to future‐proof restoration whilst still ensuring adequate genetic diversity is present in populations to allow future adaptation. This should be balanced against the need for climate‐adjusted provenance based on individual trajectories; Nimbs change for recipient estuaries (e.g., as determined by Scanes, Scanes, and Ross 2020 for eastern Australia).

Despite recent calls for studies that provide genomic data for selection of optimal seagrass donor material for use in transplantation projects (Pazzaglia et al. 2021a), little ground has been gained in this space. The present study is the first to provide a seagrass ‘donor registry’, a set of thermally pre‐adapted genotypes for use in future‐proofing eastern Australian estuaries, many of which are warming an order of magnitude faster than predicted by global models (Scanes, Scanes, and Ross 2020). Utilising this knowledge in restoration programs could bolster the success of restoration projects and provide greater climate‐resilience to this threatened yet critical habitat.

## Author Contributions

**Matt J. Nimbs:** conceptualization (lead), data curation (lead), formal analysis (lead), investigation (lead), methodology (lead), project administration (lead), resources (supporting), validation (lead), visualization (lead), writing – original draft (lead), writing – review and editing (lead). **Tim M. Glasby:** conceptualization (supporting), formal analysis (supporting), writing – review and editing (supporting). **Elizabeth A. Sinclair:** formal analysis (supporting), methodology (supporting), writing – original draft (supporting), writing – review and editing (supporting). **Daniel Swadling:** conceptualization (supporting), data curation (equal), formal analysis (supporting), writing – review and editing (supporting). **Tom R. Davis:** data curation (equal), writing – review and editing (supporting). **Melinda A. Coleman:** conceptualization (equal), formal analysis (equal), funding acquisition (lead), investigation (equal), methodology (equal), project administration (equal), resources (equal), supervision (lead), validation (equal), visualization (equal), writing – original draft (equal), writing – review and editing (equal).

## Conflicts of Interest

The authors declare no conflicts of interest.

## Data Availability

The data that support the findings of this study are openly available in Dryad at DOI: 10.5061/dryad.d2547d89s.

## References

[ece370667-bib-0001] Ahrens, C. W. , M. Byrne , and P. D. Rymer . 2019. “Standing Genomic Variation Within Coding and Regulatory Regions Contributes to the Adaptive Capacity to Climate in a Foundation Tree Species.” Molecular Ecology 28, no. 10: 2502–2516. 10.1111/mec.15092. 30950536

[ece370667-bib-0002] Amri, K. , S. Mashoreng , D. Priosambodo , N. Nurdin , and M. Lanuru . 2021. “Impact of Water Turbidity to Seagrass ( *Enhalus acoroides* ) Morphology.” IOP Conference Series: Earth and Environmental Science 860: 012020. 10.1088/1755-1315/860/1/012020.

[ece370667-bib-0003] Arnaud‐Haond, S. , C. M. Duarte , E. Diaz‐Almela , N. Marbà , T. Sintes , and E. A. Serrão . 2012. “Implications of Extreme Life Span in Clonal Organisms: Millenary Clones in Meadows of the Threatened Seagrass *Posidonia oceanica* .” PLoS One 7, no. 2: e30454. 10.1371/journal.pone.0030454. 22312426 PMC3270012

[ece370667-bib-0004] Ashizawa, D. , and J. J. Cole . 1994. “Long‐Term Temperature Trends of the Hudson River: A Study of the Historical Data.” Estuaries 17: 166–171.

[ece370667-bib-0005] Barkan, A. , and I. Small . 2014. “Pentatricopeptide Repeat Proteins in Plants.” Annual Review of Plant Biology 65, no. 1: 415–442. 10.1146/annurev-arplant-050213-040159. 24471833

[ece370667-bib-1002] Bartenfelder, A. , W. J. Kenworthy , B. Puckett , C. Deaton , and J. C. Jarvis . 2022. “The Abundance and Persistence of Temperate and Tropical Seagrasses at Their Edge‐of‐Range in the Western Atlantic Ocean.” Frontiers in Marine Science 9: 917237.

[ece370667-bib-0006] Benito‐Garzón, M. , M. Ha‐Duong , N. Frascaria‐Lacoste , and J. Fernández‐Manjarrés . 2013. “Habitat Restoration and Climate Change: Dealing With Climate Variability, Incomplete Data, and Management Decisions With Tree Translocations.” Restoration Ecology 21, no. 5: 530–536.

[ece370667-bib-0007] Benjamini, Y. , and Y. Hochberg . 1995. “Controlling the False Discovery Rate: A Practical and Powerful Approach to Multiple Testing.” Journal of the Royal Statistical Society: Series B: Methodological 57, no. 1: 289–300.

[ece370667-bib-0010] Brookes, A. J. 1999. “The Essence of SNPs.” Gene 234, no. 2: 177–186. 10395891 10.1016/s0378-1119(99)00219-x

[ece370667-bib-0011] Bürger, R. , and M. Lynch . 1995. “Evolution and Extinction in a Changing Environment: A Quantitative‐Genetic Analysis.” Evolution 49, no. 1: 151–163. 28593664 10.1111/j.1558-5646.1995.tb05967.x

[ece370667-bib-0013] Capblancq, T. , M. C. Fitzpatrick , R. A. Bay , M. Exposito‐Alonso , and S. R. Keller . 2020. “Genomic Prediction of (Mal) Adaptation Across Current and Future Climatic Landscapes.” Annual Review of Ecology, Evolution, and Systematics 51, no. 1: 245–269. 10.1146/annurev-ecolsys-020720-042553.

[ece370667-bib-0014] Cayuela, H. , Y. Dorant , C. Mérot , et al. 2021. “Thermal Adaptation Rather Than Demographic History Drives Genetic Structure Inferred by Copy Number Variants in a Marine Fish.” Molecular Ecology 30, no. 7: 1624–1641. 33565147 10.1111/mec.15835

[ece370667-bib-0015] Clark, K. , I. Karsch‐Mizrachi , D. J. Lipman , J. Ostell , and E. W. Sayers . 2016. “GenBank.” Nucleic Acids Research 44, no. D1: D67–D72. 10.1093/nar/gkv1276. 26590407 PMC4702903

[ece370667-bib-0016] Coleman, M. A. , G. Wood , K. Filbee‐Dexter , et al. 2020. “Restore or Redefine: Future Trajectories for Restoration.” Frontiers in Marine Science 7: 237.

[ece370667-bib-0019] den Hartog, C. , and J. Kuo . 2006. “Taxonomy and Biogeography of Seagrasses.” In Seagrasses: Biology, Ecology and Conservation, edited by A. W. D. Larkum , R. J. Orth , and C. M. Duarte . Dordrecht, The Netherlands: Springer.

[ece370667-bib-0020] Dey, R. , S. C. Lewis , J. M. Arblaster , and N. J. Abram . 2019. “A Review of Past and Projected Changes in Australia's Rainfall.” WIREs Climate Change 10, no. 3: e577. 10.1002/wcc.577.

[ece370667-bib-0021] Díaz‐Almela, E. , N. Marba , R. Martínez , R. Santiago , and C. M. Duarte . 2009. “Seasonal Dynamics of *Posidonia oceanica* in Magalluf Bay (Mallorca, Spain): Temperature Effects on Seagrass Mortality.” Limnology and Oceanography 54, no. 6: 2170–2182.

[ece370667-bib-0022] Dormann, C. F. , J. Elith , S. Bacher , et al. 2013. “Collinearity: A Review of Methods to Deal With It and a Simulation Study Evaluating Their Performance.” Ecography 36, no. 1: 27–46.

[ece370667-bib-0023] Duarte, B. , I. Martins , R. Rosa , et al. 2018. “Climate Change Impacts on Seagrass Meadows and Macroalgal Forests: An Integrative Perspective on Acclimation and Adaptation Potential.” Frontiers in Marine Science 5: 190.

[ece370667-bib-0024] Dunic, J. C. , C. J. Brown , R. M. Connolly , M. P. Turschwell , and I. M. Côté . 2021. “Long‐Term Declines and Recovery of Meadow Area Across the world's Seagrass Bioregions.” Global Change Biology 27, no. 17: 4096–4109. 10.1111/gcb.15684. 33993580

[ece370667-bib-0025] Dyer, K. R. 2021. “Response of Estuaries to Climate Change.” In Climate ChangeImpact on Coastal Habitation, 85–110. Boca Raton, USA: CRC Press.

[ece370667-bib-0026] Eckert, C. G. , K. E. Samis , and S. C. Lougheed . 2008. “Genetic Variation Across species' Geographical Ranges: The Central–Marginal Hypothesis and Beyond.” Molecular Ecology 17, no. 5: 1170–1188. 18302683 10.1111/j.1365-294X.2007.03659.x

[ece370667-bib-0027] Edet, O. U. , Y. S. A. Gorafi , S. Nasuda , and H. Tsujimoto . 2018. “DArTseq‐Based Analysis of Genomic Relationships Among Species of Tribe Triticeae.” Scientific Reports 8, no. 1: 1–11. 30401925 10.1038/s41598-018-34811-y PMC6219600

[ece370667-bib-0028] Edgeloe, J. M. , A. A. Severn‐Ellis , P. E. Bayer , et al. 2022. “Extensive Polyploid Clonality Was a Successful Strategy for Seagrass to Expand Into a Newly Submerged Environment.” Proceedings of the Royal Society B: Biological Sciences 289, no. 1976: 20220538. 10.1098/rspb.2022.0538. PMC9156900 35642363

[ece370667-bib-0029] Ehret, G. B. , D. Lamparter , C. J. Hoggart , J. C. Whittaker , J. S. Beckmann , and Z. Kutalik . 2012. “A Multi‐SNP Locus‐Association Method Reveals a Substantial Fraction of the Missing Heritability.” American Journal of Human Genetics 91, no. 5: 863–871. 10.1016/j.ajhg.2012.09.013. 23122585 PMC3487134

[ece370667-bib-0030] Ellis, N. , S. J. Smith , and C. R. Pitcher . 2012. “Gradient Forests: Calculating Importance Gradients on Physical Predictors.” Ecology 93, no. 1: 156–168. 22486096 10.1890/11-0252.1

[ece370667-bib-0031] Evans, S. M. , E. A. Sinclair , A. G. B. Poore , K. F. Bain , and A. Vergés . 2018. “Assessing the Effect of Genetic Diversity on the Early Establishment of the Threatened Seagrass *Posidonia australis* Using a Reciprocal‐Transplant Experiment.” Restoration Ecology 26, no. 3: 570–580.

[ece370667-bib-0032] Evans, S. M. , E. A. Sinclair , A. G. B. Poore , P. D. Steinberg , G. A. Kendrick , and A. Vergés . 2014. “Genetic Diversity in Threatened *Posidonia australis* Seagrass Meadows.” Conservation Genetics 15: 717–728.

[ece370667-bib-0033] Fitzpatrick, M. C. , and S. R. Keller . 2015. “Ecological Genomics Meets Community‐Level Modelling of Biodiversity: Mapping the Genomic Landscape of Current and Future Environmental Adaptation.” Ecology Letters 18, no. 1: 1–16. 25270536 10.1111/ele.12376

[ece370667-bib-0035] Frichot, E. , and O. François . 2015. “LEA: An R Package for Landscape and Ecological Association Studies.” Methods in Ecology and Evolution 6, no. 8: 925–929.

[ece370667-bib-0036] Frichot, E. , S. D. Schoville , G. Bouchard , and O. François . 2013. “Testing for Associations Between Loci and Environmental Gradients Using Latent Factor Mixed Models.” Molecular Biology and Evolution 30, no. 7: 1687–1699. 23543094 10.1093/molbev/mst063 PMC3684853

[ece370667-bib-0037] Fuggle, R. E. , P. E. Gribben , and E. M. Marzinelli . 2023. “Experimental Evidence Root‐ Associated Microbes Mediate Seagrass Response to Environmental Stress.” Journal of Ecology 111: 1079–1093.

[ece370667-bib-0038] Fulton, E. A. , N. Mazloumi , A. Puckeridge , and R. Hanamseth . 2024. “Modelling Perspective on the Climate Footprint in South East Australian Marine Waters and Its Fisheries.” ICES Journal of Marine Science 81: 130–144.

[ece370667-bib-0039] Gillanders, B. M. , T. S. Elsdon , I. A. Halliday , G. P. Jenkins , J. B. Robins , and F. J. Valesini . 2011. “Potential Effects of Climate Change on Australian Estuaries and Fish Utilising Estuaries: A Review.” Marine and Freshwater Research 62, no. 9: 1115–1131.

[ece370667-bib-0040] Grech, A. , K. Chartrand‐Miller , P. Erftemeijer , et al. 2012. “A Comparison of Threats, Vulnerabilities, and Management Approaches in Global Seagrass Bioregions.” Environmental Research Letters 7, no. 2: 024006.

[ece370667-bib-0041] Gruber, B. , P. Unmack , O. Berry , and A. Georges . 2019. Introduction to dartR. Canberra, Australia: CRAN.

[ece370667-bib-0042] Guerrero‐Meseguer, L. , T. E. Cox , C. Sanz‐Lázaro , et al. 2020. “Does Ocean Acidification Benefit Seagrasses in a Mesohaline Environment? A Mesocosm Experiment in the Northern Gulf of Mexico.” Estuaries and Coasts 43: 1377–1393.

[ece370667-bib-0043] Hedrick, P. W. 2012. “What Is the Evidence for Heterozygote Advantage Selection?” Trends in Ecology & Evolution 27, no. 12: 698–704. 22975220 10.1016/j.tree.2012.08.012

[ece370667-bib-0044] Hobday, A. J. , and G. T. Pecl . 2014. “Identification of Global Marine Hotspots: Sentinels for Change and Vanguards for Adaptation Action.” Reviews in Fish Biology and Fisheries 24: 415–425.

[ece370667-bib-0045] Hoffmann, A. A. , and C. M. Sgrò . 2011. “Climate Change and Evolutionary Adaptation.” Nature 470, no. 7335: 479–485. 10.1038/nature09670. 21350480

[ece370667-bib-0046] Hogarth, P. J. 2007. “The Biology of Mangroves and Seagrasses.” In The Biology of Habitat Series. New York, NY, USA: Oxford University Press.

[ece370667-bib-0047] Holland, O. J. , C. Smythe , T. D. Clark , et al. 2024. “Size‐Dependent Thermal Limits in Australian Hybrid Abalone: Implications for Productivity Shifts With Ocean Warming.” Reviews in Fish Biology and Fisheries 34: 271–291.

[ece370667-bib-0049] Huntley, R. P. , T. Sawford , P. Mutowo‐Meullenet , et al. 2015. “The GOA Database: Gene Ontology Annotation Updates for 2015.” Nucleic Acids Research 43, no. D1: D1057–D1063. 10.1093/nar/gku1113. 25378336 PMC4383930

[ece370667-bib-0050] Inglis, G. J. , and M. P. L. Smith . 1998. “Synchronous Flowering of Estuarine Seagrass Meadows.” Aquatic Botany 60, no. 1: 37–48.

[ece370667-bib-0051] Invers, O. , J. Romero , and M. Pérez . 1997. “Effects of pH on Seagrass Photosynthesis: A Laboratory and Field Assessment.” Aquatic Botany 59, no. 3–4: 185–194.

[ece370667-bib-0052] Jaccoud, D. , K. Peng , D. Feinstein , and A. Kilian . 2001. “Diversity Arrays: A Solid State Technology for Sequence Information Independent Genotyping.” Nucleic Acids Research 29, no. 4: e25. 11160945 10.1093/nar/29.4.e25 PMC29632

[ece370667-bib-0053] Johnson, M. , I. Zaretskaya , Y. Raytselis , Y. Merezhuk , S. McGinnis , and T. L. Madden . 2008. “NCBI BLAST: A Better Web Interface.” Nucleic Acids Research 36, no. suppl_2: W5–W9. 18440982 10.1093/nar/gkn201 PMC2447716

[ece370667-bib-0054] Jongen, R. , E. M. Marzinelli , A. B. Bugnot , et al. 2024. “Integrating Belowground Interactions Into Seagrass Restoration Strategies.” Oceanography and Marine Biology: An Annual Review 62: 192–214.

[ece370667-bib-0056] Kamvar, Z. N. , J. F. Tabima , and N. J. Grunwald . 2014. “Poppr: An R Package for Genetic Analysis of Populations With Clonal, Partially Clonal, and/or Sexual Reproduction.” PeerJ 2013: 1–14. 10.7717/peerj.281 PMC3961149 24688859

[ece370667-bib-0057] Kilian, A. , P. Wenzl , E. Huttner , et al. 2012. “Diversity Arrays Technology: A Generic Genome Profiling Technology on Open Platforms.” In Data Production and Analysis in Population Genomics. Methods in Molecular Biology (Methods and Protocols), 67–89. Clifton, NJ, USA: Humana Press. 10.1007/978-1-61779-870-2_5 22665276

[ece370667-bib-0058] Kilminster, K. , K. McMahon , M. Waycott , et al. 2015. “Unravelling Complexity in Seagrass Systems for Management: Australia as a Microcosm.” Science of the Total Environment 534: 97–109. 25917445 10.1016/j.scitotenv.2015.04.061

[ece370667-bib-0059] Krauss, S. L. , E. A. Sinclair , J. D. Bussell , and R. J. Hobbs . 2013. “An Ecological Genetic Delineation of Local Seed‐Source Provenance for Ecological Restoration.” Ecology and Evolution 3, no. 7: 2138–2149. 10.1002/ece3.595. 23919158 PMC3728953

[ece370667-bib-1003] Larkum, A. W. D. 1976. “Ecology of Botany Bay. I. Growth of Posidonia australis (Brown) Hook. F. in Botany Bay and Other Bays of the Sydney Basin.” Marine and Freshwater Research 27, no. 1: 117–127.

[ece370667-bib-0060] Legendre, P. , and M. De Cáceres . 2013. “Beta Diversity as the Variance of Community Data: Dissimilarity Coefficients and Partitioning.” Ecology Letters 16, no. 8: 951–963. 10.1111/ele.12141. 23809147

[ece370667-bib-0061] Legendre, P. , and L. Legendre . 2012. Numerical Ecology. Oxford, UK: Elsevier.

[ece370667-bib-0062] Lekammudiyanse, M. U. , M. I. Saunders , N. Flint , et al. 2024. “Environmental Drivers of Flowering in the Genus *Zostera* and Spatio‐Temporal Variability of *Zostera muelleri* Flowering in Australasia.” Aquatic Conservation: Marine and Freshwater Ecosystems 34: e4068.

[ece370667-bib-0063] Luu, K. , E. Bazin , and M. G. B. Blum . 2017. “Pcadapt: An R Package to Perform Genome Scans for Selection Based on Principal Component Analysis.” Molecular Ecology Resources 17, no. 1: 67–77. 27601374 10.1111/1755-0998.12592

[ece370667-bib-0064] Marbà, N. , and C. M. Duarte . 2010. “Mediterranean Warming Triggers Seagrass ( *Posidonia oceanica* ) Shoot Mortality.” Global Change Biology 16, no. 8: 2366–2375.

[ece370667-bib-0066] McMahon, K. , K. Kilminster , R. Canto , et al. 2022. “The Risk of Multiple Anthropogenic and Climate Change Threats Must Be Considered for Continental Scale Conservation and Management of Seagrass Habitat.” Frontiers in Marine Science 9: 837259.

[ece370667-bib-0067] Mijangos, J. L. , B. Gruber , O. Berry , C. Pacioni , and A. Georges . 2022. “dartR v2: An Accessible Genetic Analysis Platform for Conservation, Ecology and Agriculture.” Methods in Ecology and Evolution 13, no. 10: 2150–2158. 10.1111/2041-210X.13918.

[ece370667-bib-0068] Nguyen, H. M. , M. Ruocco , E. Dattolo , et al. 2023. “Signs of Local Adaptation by Genetic Selection and Isolation Promoted by Extreme Temperature and Salinity in the Mediterranean Seagrass *Posidonia oceanica* .” Molecular Ecology 32, no. 15: 4313–4328. 37271924 10.1111/mec.17032

[ece370667-bib-0070] Oczkowski, A. , R. McKinney , S. Ayvazian , A. Hanson , C. Wigand , and E. Markham . 2015. “Preliminary Evidence for the Amplification of Global Warming in Shallow, Intertidal Estuarine Waters.” PLoS One 10, no. 10: e0141529. 26510009 10.1371/journal.pone.0141529 PMC4624981

[ece370667-bib-0071] Oksanen, J. , F. G. Blanchet , R. Kindt , et al. 2013. “Package ‘Vegan’.” Community Ecology Package, Version 2, no. 9: 1–295.

[ece370667-bib-0072] Oliver, E. C. J. , M. T. Burrows , M. G. Donat , et al. 2019. “Projected Marine Heatwaves in the 21st Century and the Potential for Ecological Impact.” Frontiers in Marine Science 6: 734. 10.3389/fmars.2019.00734.

[ece370667-bib-0073] Orgogozo, V. , B. Morizot , and A. Martin . 2015. “The Differential View of Genotype–Phenotype Relationships.” Frontiers in Genetics 6: 144986. 10.3389/fgene.2015.00179 PMC4437230 26042146

[ece370667-bib-0074] Page, M. J. , and E. Di Cera . 2008. “Serine Peptidases: Classification, Structure and Function.” Cellular and Molecular Life Sciences 65, no. 7–8: 1220–1236. 10.1007/s00018-008-7565-9. 18259688 PMC11131664

[ece370667-bib-0075] Pazzaglia, J. , H. M. Nguyen , A. Santillán‐Sarmiento , et al. 2021a. “The Genetic Component of Seagrass Restoration: What We Know and the Way Forwards.” Watermark 13, no. 6: 829.

[ece370667-bib-0076] Pazzaglia, J. , A. Santillán‐Sarmiento , E. Dattolo , et al. 2021b. “Integrative Responses of *Posidonia oceanica* to Multiple Stressors: A New Prospective for Future Global Changes.”

[ece370667-bib-0077] Phair, N. 2016. Vulnerability to Future Environmental Conditions and Population Genetics of the Seagrass, Zostera capensis. Stellenbosch, South Africa: University of Stellenbosch.

[ece370667-bib-0078] Phair, N. L. , R. J. Toonen , I. Knapp , and S. von der Heyden . 2019. “Shared Genomic Outliers Across Two Divergent Population Clusters of a Highly Threatened Seagrass.” PeerJ 7: e6806. 31106053 10.7717/peerj.6806 PMC6497040

[ece370667-bib-0079] Privé, F. , K. Luu , B. J. Vilhjálmsson , and M. G. B. Blum . 2020. “Performing Highly Efficient Genome Scans for Local Adaptation With R Package Pcadapt Version 4.” Molecular Biology and Evolution 37, no. 7: 2153–2154. 32343802 10.1093/molbev/msaa053

[ece370667-bib-0080] Prober, S. M. , M. Byrne , E. H. McLean , et al. 2015. “Climate‐Adjusted Provenancing: A Strategy for Climate‐Resilient Ecological Restoration.” Frontiers in Ecology and Evolution 3: 65.

[ece370667-bib-0081] Prober, S. M. , V. A. J. Doerr , L. M. Broadhurst , K. J. Williams , and F. Dickson . 2019. “Shifting the Conservation Paradigm: A Synthesis of Options for Renovating Nature Under Climate Change.” Ecological Monographs 89, no. 1: e01333.

[ece370667-bib-0082] Procaccini, G. , and L. Piazzi . 2001. “Genetic Polymorphism and Transplantation Success in the Mediterranean Seagrass *Posidonia oceanica* .” Restoration Ecology 9, no. 3: 332–338.

[ece370667-bib-0083] Quintero, I. , and J. J. Wiens . 2013. “Rates of Projected Climate Change Dramatically Exceed Past Rates of Climatic Niche Evolution Among Vertebrate Species.” Ecology Letters 16, no. 8: 1095–1103. 23800223 10.1111/ele.12144

[ece370667-bib-0084] Reusch, T. B. H. , I. B. Baums , and B. Werner . 2021. “Evolution via Somatic Genetic Variation in Modular Species.” Trends in Ecology & Evolution 36, no. 12: 1083–1092. 34538501 10.1016/j.tree.2021.08.011

[ece370667-bib-0085] Reynolds, L. K. , K. J. McGlathery , and M. Waycott . 2012. “Genetic Diversity Enhances Restoration Success by Augmenting Ecosystem Services.” PLoS One 7, no. 6: e38397. 22761681 10.1371/journal.pone.0038397 PMC3382623

[ece370667-bib-0086] Rossetto, M. , J. Bragg , A. Kilian , H. McPherson , M. van der Merwe , and P. D. Wilson . 2019. “Restore and Renew: A Genomics‐Era Framework for Species Provenance Delimitation.” Restoration Ecology 27, no. 3: 538–548.

[ece370667-bib-0087] Ruan, X. , Z. Wang , Y. Su , and T. Wang . 2021. “Population Genomics Reveals Gene Flow and Adaptive Signature in Invasive Weed *Mikania micrantha* .” Genes 12, no. 8: 1279. 10.3390/genes12081279. 34440453 PMC8394975

[ece370667-bib-0088] Ruocco, M. , M. Jahnke , J. Silva , G. Procaccini , and E. Dattolo . 2022. “2b‐RAD Genotyping of the Seagrass Cymodocea Nodosa Along a Latitudinal Cline Identifies Candidate Genes for Environmental Adaptation.” Frontiers in Genetics 13: 866758. 35651946 10.3389/fgene.2022.866758 PMC9149362

[ece370667-bib-0089] Said, N. , C. Webster , N. Dunham , S. Strydom , and K. McMahon . 2024. “Seagrass Thermal Tolerance Varies Between Species and Within Species Across Locations.” Prepared for the WAMSI Westport Marine Science Program. Western Australian Marine Science Institution, Perth, Western Australia, 26 pp.

[ece370667-bib-0090] Sansaloni, C. , C. Petroli , D. Jaccoud , et al. 2011. “Diversity Arrays Technology (DArT) and Next‐Generation Sequencing Combined: Genome‐Wide, High Throughput, Highly Informative Genotyping for Molecular Breeding of Eucalyptus.” BMC Proceedings 5, no. 7: 1–2.

[ece370667-bib-0091] Scanes, E. , P. R. Scanes , and P. M. Ross . 2020. “Climate Change Rapidly Warms and Acidifies Australian Estuaries.” Nature Communications 11, no. 1: 1803. 10.1038/s41467-020-15550-z PMC7156424 32286277

[ece370667-bib-0092] Schiebelhut, L. M. , R. K. Grosberg , J. J. Stachowicz , and R. A. Bay . 2023. “Genomic Responses to Parallel Temperature Gradients in the Eelgrass *Zostera marina* in Adjacent Bays.” Molecular Ecology 32, no. 11: 2835–2849. 10.1111/mec.16899. 36814144

[ece370667-bib-0093] Short, F. , T. Carruthers , W. Dennison , and M. Waycott . 2007. “Global Seagrass Distribution and Diversity: A Bioregional Model.” Journal of Experimental Marine Biology and Ecology 350, no. 1–2: 3–20.

[ece370667-bib-0094] Sinclair, E. A. , R. K. Hovey , S. L. Krauss , J. M. Anthony , M. Waycott , and G. A. Kendrick . 2023. “Historic and Contemporary Biogeographic Perspectives on Range‐Wide Spatial Genetic Structure in a Widespread Seagrass.” Ecology and Evolution 13, no. 3: e9900. 10.1002/ece3.9900. 36950371 PMC10025079

[ece370667-bib-0096] State Government of NSW and NSW Department of Climate Change, E. the E. and W . 2024. “State Government of NSW and NSW Department of Climate Change, Energy, the Environment and Water 2024, NSW Estuary Water Quality Data Compilation: 2007–2020, Accessed From the Sharing and Enabling Environmental Data Portal.”

[ece370667-bib-1001] Strydom, S. , K. Murray , S. Wilson , et al. 2020. “Too Hot to Handle: Unprecedented Seagrass Death Driven by Marine Heatwave in a World Heritage Area.” Global Change Biology 26, no. 6: 3525–3538. 32129909 10.1111/gcb.15065

[ece370667-bib-0097] Swadling, D. S. , G. J. West , P. T. Gibson , R. J. Laird , and T. M. Glasby . 2023a. “Don't Go Breaking Apart: Anthropogenic Disturbances Predict Meadow Fragmentation of an Endangered Seagrass.” Aquatic Conservation: Marine and Freshwater Ecosystems 33, no. 1: 56–69.

[ece370667-bib-0098] Swadling, D. S. , G. J. West , P. T. Gibson , R. J. Laird , and T. M. Glasby . 2023b. “Multi‐Scale Assessments Reveal Changes in the Distribution of the Endangered Seagrass *Posidonia australis* and the Role of Disturbances.” Marine Biology 170, no. 11: 147.

[ece370667-bib-0099] Tan, Y. M. , O. Dalby , G. A. Kendrick , et al. 2020. “Seagrass Restoration Is Possible: Insights and Lessons From Australia and New Zealand.” Frontiers in Marine Science 7: 617. 10.3389/fmars.2020.00617.

[ece370667-bib-0101] Tomas, F. , G. Hernan , J. Mañez‐Crespo , et al. 2024. “Mass Flowering and Unprecedented Extended Pseudovivipary in Seagrass ( *Posidonia oceanica* ) After a Marine Heat Wave.” Marine Pollution Bulletin 203: 116394. 10.1016/j.marpolbul.2024.116394. 38705001

[ece370667-bib-0102] University of Technology Sydney , NSW Department of Primary Industries and Regional Development (DPIRD) , The Yield Hunter Local Land Services , and NSW Oyster Farmers . 2024. “Australian Shellfish Transformation Project Data Portal.”

[ece370667-bib-0103] Unsworth, R. K. F. , L. M. Nordlund , and L. C. Cullen‐Unsworth . 2019. “Seagrass Meadows Support Global Fisheries Production.” Conservation Letters 12: e12566.

[ece370667-bib-0104] Urban, S. 2006. “Rhomboid Proteins: Conserved Membrane Proteases With Divergent Biological Functions.” Genes & Development 20, no. 22: 3054–3068. 17114579 10.1101/gad.1488606

[ece370667-bib-0105] Vranken, S. , T. Wernberg , A. Scheben , et al. 2021. “Genotype–Environment Mismatch of Kelp Forests Under Climate Change.” Molecular Ecology 30, no. 15: 3730–3746. 34018645 10.1111/mec.15993

[ece370667-bib-0107] Walker, L. D. A. , P. E. Gribben , T. M. Glasby , E. M. Marzinelli , D. R. Varkey , and K. A. Dafforn . 2024. “Above and Below‐Ground Bacterial Communities Shift in Seagrass Beds With Warming Temperatures.” Frontiers in Marine Science 11: 1374946.

[ece370667-bib-0108] Wang, L. , Y. Ji , Y. Hu , et al. 2019. “The Architecture of Intra‐Organism Mutation Rate Variation in Plants.” PLoS Biology 17, no. 4: e3000191. 30964866 10.1371/journal.pbio.3000191 PMC6456163

[ece370667-bib-0109] Waycott, M. , C. M. Duarte , T. J. B. Carruthers , et al. 2009. “Accelerating Loss of Seagrasses Across the Globe Threatens Coastal Ecosystems.” Proceedings of the National Academy of Sciences of the United States of America 106, no. 30: 12377–12381. 19587236 10.1073/pnas.0905620106 PMC2707273

[ece370667-bib-0110] Webster, M. M. , B. Twohey , P. S. Alagona , et al. 2023. “Assisting Adaptation in a Changing World.” Frontiers in Environmental Science 11: 1232374.

[ece370667-bib-0112] Wernberg, T. , M. S. Thomsen , J. K. Baum , et al. 2024. “Impacts of Climate Change on Marine Foundation Species.” Annual Review of Marine Science 16, no. 1: 247–282. 10.1146/annurev-marine-042023-093037. 37683273

[ece370667-bib-0113] West, G. J. , and T. M. Glasby . 2022. “Interpreting Long‐Term Patterns of Seagrasses Abundance: How Seagrass Variability Is Dependent on Genus and Estuary Type.” Estuaries and Coasts 45, no. 5: 1393–1408.

[ece370667-bib-0114] West, R. J. , A. W. D. Larkum , and R. J. King . 1989. “Regional Studies—Seagrasses of South Eastern Australia.” In Biology of Seagrasses. A Treatise on the Biology of Seagrasses With Special Reference to the Australian Region, edited by A. W. D. Larkum , A. J. McComb , and S. A. Shepherd , 230–260. Amsterdam: Elsevier Science Publishers BV.

[ece370667-bib-0115] Williams, A. N. , S. Ulm , T. Sapienza , S. Lewis , and C. S. M. Turney . 2018. “Sea‐Level Change and Demography During the Last Glacial Termination and Early Holocene Across the Australian Continent.” Quaternary Science Reviews 182: 144–154.

[ece370667-bib-0116] Wiltshire, K. H. , and B. F. J. Manly . 2004. “The Warming Trend at Helgoland Roads, North Sea: Phytoplankton Response.” Helgoland Marine Research 58: 269–273.

[ece370667-bib-0117] Winder, R. , E. Nelson , and T. Beardmore . 2011. “Ecological Implications for Assisted Migration in Canadian Forests.” Forestry Chronicle 87, no. 6: 731–744.

[ece370667-bib-0118] Wood, G. , E. M. Marzinelli , M. A. Coleman , et al. 2019. “Restoring Subtidal Marine Macrophytes in the Anthropocene: Trajectories and Future‐Proofing.” Marine and Freshwater Research 70, no. 7: 936–951.

[ece370667-bib-0119] Wood, G. V. , K. J. Griffin , M. van der Mheen , et al. 2024. “Reef Adapt: A Tool to Inform Climate‐Smart Marine Restoration and Management Decisions.” Communications Biology 7, no. 1: 1368. 39478133 10.1038/s42003-024-06970-4 PMC11526119

[ece370667-bib-0120] Yu, L. , C. Boström , S. Franzenburg , T. Bayer , T. Dagan , and T. B. H. Reusch . 2020. “Somatic Genetic Drift and Multilevel Selection in a Clonal Seagrass.” Nature Ecology & Evolution 4, no. 7: 952–962. 32393866 10.1038/s41559-020-1196-4

